# Microfabricated self-referencing pulstrodes†

**DOI:** 10.1039/d5sd00024f

**Published:** 2025-05-14

**Authors:** Ayian Speck, Davide Migliorelli, Jeremy Disser, Silvia Generelli, Guillaume Bouilly, Tara Forrest, Elena Zdrachek, Loïc Burr, Eric Bakker

**Affiliations:** a Department of Inorganic and Analytical Chemistry, University of Geneva Quai E.-Ansermet 30 1211 Geneva 4 Switzerland eric.bakker@unige.ch; b CSEM Landquart Bahnhofstrasse 1 7302 Landquart Switzerland

## Abstract

Screen printing and inkjet printing are attractive processes to produce low-cost and mass producible electroanalytical sensors. Despite important advances in the field, obtaining a printed electrochemical reference element that satisfies analytical requirements has not yet been realized satisfactorily. This paper investigates the use of screen printing and inkjet printing to produce a self-contained, all-solid state reference element that can be integrated with a wide range of electroanalytical sensing principles. The principle relies on a silver/silver iodide element that self-generates its potential by the application of a so-called pulstrode protocol. Specifically, a defined quantity of iodide is released by a short cathodic current pulse, and the reference potential defined by the released iodide is subsequently recorded at zero current. Both screen and inkjet-printed reference electrodes are fabricated and characterized, and the methodology optimized and assessed. As an application example, a single-point calibration method is used to quantify ions in undiluted filtered urine samples by potentiometry. The screen-printing approach was less successful owing to the low purity of the silver ink used. The inkjet printing approach allowed one to quantify chloride and sodium in urine. Using a conventional silver/silver chloride reference electrode as standard, relative errors of respectively 7.7 and 14.1% for chloride and sodium were obtained. While the approach would benefit from further optimization for long term applications, especially the use of high purity silver inks, it is a promising strategy for the realization of fully integrated all-solid-state microfabricated sensing systems.

## Abstract 2

Microfabricated self-referencing elements based on the pulstrodes principle are realized and integrated into electrochemical sensors that no longer require traditional reference electrodes.

## Graphical abstract

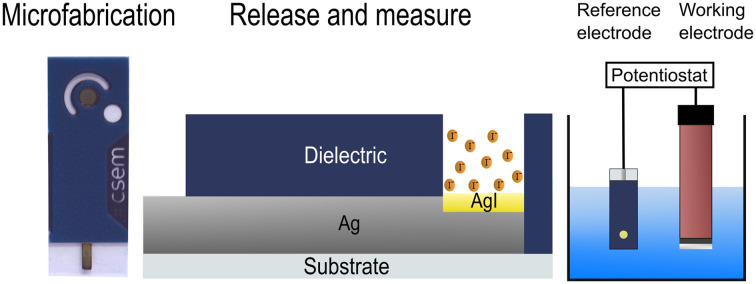

## Introduction

The healthcare sector requires diagnostic tools to reliably and rapidly measure health parameters at the point-of-care. In this regard, electrochemical sensors, including wearable and point-of-care testing devices play a crucial role.^1,2^ These sensors offer reliable, rapid, cost-effective, precise, and continuous or semi-continuous measurements, allowing efficient monitoring of various health parameters and metabolites.^3–5^

While significant progress has been achieved regarding sensing principles and materials, it is equally important to address the electrochemical reference element. The reference electrode must provide a stable and constant potential that is reasonably independent of the sample composition. Unfortunately, traditional reference electrodes require a voluminous electrolyte reservoir connected to the sample through a liquid junction and are therefore bulky and difficult to miniaturize. Dynamic electrochemical techniques such as amperometry or voltammetry^6–8^ may tolerate reference elements with less stringent requirements since current is the output signal. However, potentiometric probes cannot afford the use of pseudo-reference electrodes of questionable quality since the potential is directly related to the desired sample activity/concentration. Recent research has therefore explored so-called liquid junction-free reference electrodes, particularly solid-state reference electrodes (SSREs).

Arguably the most widely applied SSREs consist of a polymeric membrane doped with a chloride salt, for example KCl in a vinyl-ester resin^9^ or NaCl in polyvinyl butyral.^10^ Girault *et al.* proposed the use of KCl in a polyacrylate UV-curable ink^11^ that yields flexible SSREs in a relatively simple fabrication process. The number of SSREs based on chloride salts reported in the literature demonstrates their interest as a reference element. However, one common drawback is their high electrical resistance, and the sample must not contain any interfering ions such as sulfide, iodide or bromide.^12,13^ The leaching of the chloride salt from the polymer will result in a potential drift with time, which depends on the sample composition.

Another example includes reference electrodes using moderately lipophilic electrolytes (ionic liquids) and other lipophilic salts. It is known that the phase boundary potential is kept constant by the partitioning of the electrolyte from the reference material into the sample, independent of its concentration. Such reference elements exhibit a high electrical conductivity and long term stability.^14^ Zhang *et al.*^15^ proposed in 2012 an ionic liquid-based RE with excellent long term stability, exhibiting an EMF drift of 0.042 mV h^−1^ over 26 days in a high chloride background. However, ionic liquid-based reference elements manifest drawbacks such as their eventual loss into the sample, limiting miniaturizability, and the risk of contamination of neighboring polymeric sensing elements that can result in signal deterioration.^16–18^ Low reproducibility of the ionic-liquid based reference electrode is also of concern: Linder and co-workers reported that this variability is largely due to the lack of stoichiometry regarding the ionic liquid cation and anion,^19^ often not present in a perfect 1 : 1 ratio due to impurities in the salts. Lipophilic sample ions can also interfere. Tiuftiakov *et al.*^20^ recently introduced a model that accounts for ion pairing in the polymeric phase in addition to ion-exchange/co-extraction equilibria. This model enabled accurate predictions of the failure of the reference element beyond a certain concentration threshold of interfering sample ions.

In the context of SSREs, screen-printed electrodes (SPEs) are of great interest. They are manufactured by printing appropriate inks with a desired pattern on a flat plastic or ceramic substrate. In addition to various types of inks that can be utilized, such as gold, silver, carbon and platinum,^21^ the ink composition can be further modified with metals, enzymes and complexing agents.^22^ Their potential for miniaturization, low cost of fabrication and ease of mass production make SPEs a desirable approach to design a reference element.^23,24^ However, poor long-term stability^25^ and significant batch-to-batch variations (hence variations in *E*^0^ values)^26^ are common drawbacks of screen-printed electrodes. Dawkins *et al.*^27^ proposed in 2021 a screen-printed Ag/AgCl reference electrode integrated with KCl electrolyte and PDMS junction, which displayed minimal potential over a substantial lifetime of up to 27 days and adequate insensitivity to common interferences. However, this SPE construction comes at the cost of a rather long hydration time (40 min) and a complicated fabrication process. Mamińska *et al.*^12^ reported the fabrication of a screen-printed ionic liquid based reference electrode which exhibited good long term stability and inter-electrode potential reproducibility, but suffered from long equilibration time to reach stable potential (4 h), which is unfortunately an undesired characteristic in point-of-care applications.

Inkjet-printed electrodes (IPEs) have emerged as a versatile alternative with a number of benefits, which include a higher degree of purity of the utilized inks, resulting in less interferences, a more uniform deposition and a high bio-compatibility of the material, more precise patterning, and customizable designs.^28–30^ Moya *et al.*^31^ proposed in 2019 an IPE-RE based on a silver chloride element and a chloride-containing PVB matrix which seems promising. However, the inter-electrode *E*^0^ reproducibility is not discussed, nor is the electrode behavior in chloride solution with molarities higher than 10^−2^ M investigated. Real-world physiological applications often involve chloride backgrounds of about 10^−1^ M. The main drawbacks associated with inkjet printing are the need for a specialized and costly printing equipment and a requirement for substrate to ink compatibility. In recent years, laser induced graphene has also emerged as an attractive fabrication method in which patterning is achieved by a guided laser.^32–34^ However, this technique is not available for a wider range of electrode materials.

**Fig. 1:**
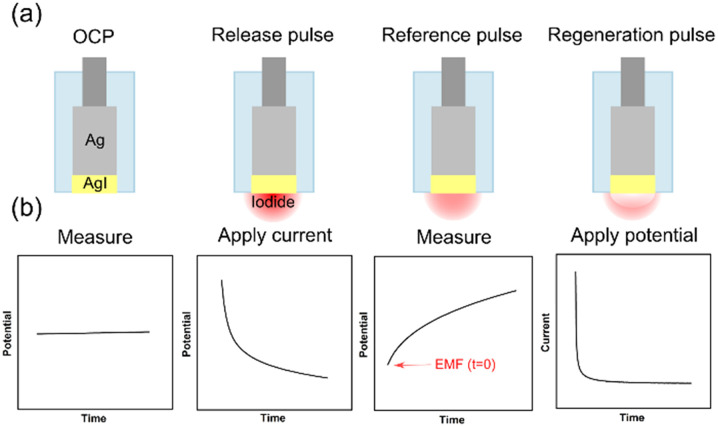
Illustration of the principle (a) and potential and current changes (b).

In 2020, our group introduced a solid-state reference electrode that relies on an Ag/AgI element and acts as a pulstrode to self-generate a reference potential.^35^ Specifically, a defined quantity of iodide is released from the electrode by the application of a cathodic current pulse. Subsequently, the open-circuit potential is measured at a predefined time and is a direct function of the released iodide at the electrode surface (Fig. 1). This self-referencing system is completely solid-state and contains no spontaneously leachable components, which sets itself apart from all other reference element principles discussed above.

This work explores the manufacturing and analytical characteristics of both screen-printed and inkjet-printed electrodes based on the self-referencing pulstrode approach. The performance of inkjet-printed electrodes as reference element is elucidated for the potentiometric detection of cationic and anionic analytes in urine samples. The monitoring of urine electrolytes such as chloride and sodium is of great clinical relevance. It allows for kidney function assessment, as abnormal levels may indicates dehydration, renal disorders, kidney disease or improper acid–base equilibrium regulation.^36,37^ Furthermore, the biological nature of urine results in physical (adsorption) and chemical (electrostatic interactions, complex formations, ion strength variability, *etc.*) interferences, which makes it a challenging matrix for potentiometry.

Using urine to demonstrate the reliability of the pulstrode protocol with inkjet-printed electrodes provides a strong basis for further applications. The work underscores the significant potential of the pulstrode approach for all-solid-state, microfabricated electrochemical sensors.

## Experimental section

### Materials and reagents

Sodium bicarbonate (NaHCO_3_), sodium chloride (NaCl), potassium chloride (KCl), potassium nitrate (KNO_3_), potassium hydroxide (KOH), creatinine, citric acid, ascorbic acid, sodium ionophore X (4-tert-butylcalix[4]arene tetraacetic acid tetraethylester, NaX), sodium tetrakis[3,5-bis(trifluoromethyl)phenyl]borate (NaTFPB), high molecular weight poly(vinyl chloride) (PVC), bis(2-ethylhexyl) sebacate (DOS), tetrakis(4-chlorophenyl)borate tetradodecylammonium salt (ETH 500), carbon nanotubes single-walled (octadecyl-amine functionalized) and tetrahydrofuran (THF) were purchased from Sigma Aldrich. Sodium iodide (NaI) and sulfuric acid were purchased from Acros Organic. 1 M hydrochloric acid (HCl) volumetric solution and monopotassium phosphate were purchased from Fisher Scientific. Urea was purchased from Fluka. Conductive inks were purchased from Oreltech and DuPont, printed dielectric was purchased from SunChemical and DuPont. Aqueous solutions were prepared by dissolving the respective salts in deionized water (>18 MΩ cm).

### Electrochemical equipment

The electrochemical measurements were carried out with a potentiostat (Lawson Labs Inc.) controlled by a personal computer using EMF16 Interface software or with a galvanostat potentiostat PGSTAT128N (Metrohm Autolab) controlled by a personal computer using Nova 2.1.4 software.

### Preparation of the electrodes

#### Macroelectrodes

**Fig. 2:**
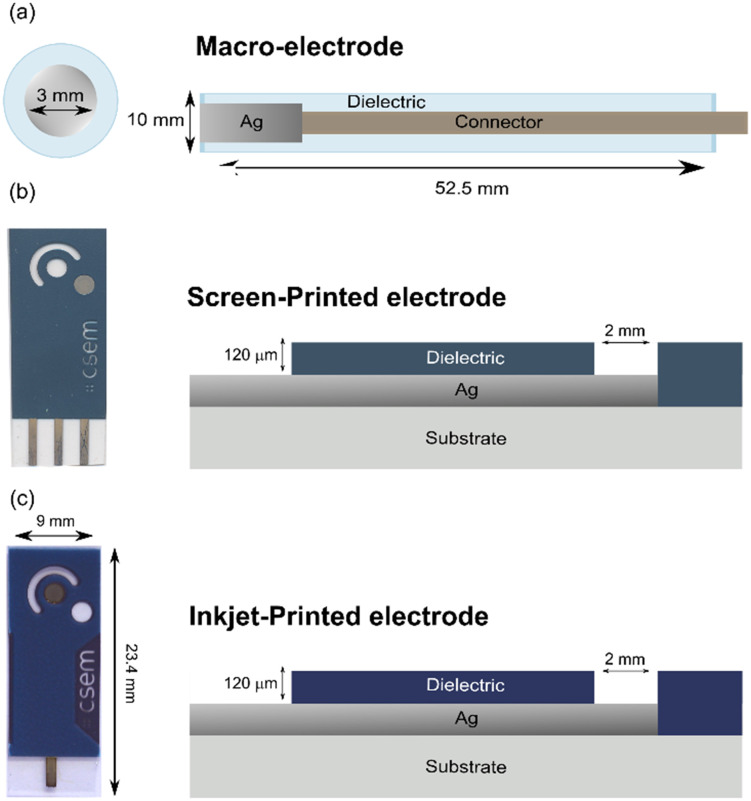
Pictures and schematic representations of the three different type of electrodes used in the present work. Commercially available macroelectrode (a). For printed electrodes, the silver electrode used as reference element is shown in grey (bottom right electrode for (b) and middle electrode for (c)). Its diameter is 2 mm.

The Ag/AgI electrode was prepared by electrochemically depositing an AgI layer on the surface of a commercial Ag electrode (Fig. 2a) (diameter of 3.0 mm, Metrohm, Switzerland) in a 0.1 M NaI solution for one hour at a constant anodic current density of 0.5 mA cm^−2^.

#### Screen-printed electrodes

The electrodes were fabricated using a standard screen-printing technique using a semi-automatic screen printer (Aurel VS1520) and stainless-steel mesh screens (Fig. 2b). The PET substrate was treated by oxygen plasma and the Ag ink was subsequently printed using an emulsion of 8–10 μm. The Ag ink was then cured according to the supplier's protocols before the printing of the dielectric layer using an emulsion of 8–10 μm and curing according to the supplier's instructions.

The Ag/AgI electrodes were prepared by electrochemically depositing an AgI layer on the surface of the screen-printed Ag electrodes (diameter 2.0 mm) in a 0.1 M NaI solution at a constant anodic current density of 0.5 mA cm^−2^ for various times.

#### Inkjet-printed electrodes

The electrodes were fabricated by inkjet printing using samba cartridges in a Ceradrop printer (MGI group). First, the PET substrate was treated by oxygen plasma (Fig. 2c). Then, the Oreltech Ag ink was printed onto the PET substrate at varying frequencies and temperature nozzles prior curing by Argon plasma and photonic treatment. The following fabrication steps for the dielectric layer and the Ag/AgCl electrodes were identical to the procedures described for the screen-printed electrodes.

#### Chloride ion-selective electrode

The Ag/AgCl electrode was prepared by electrochemically depositing an AgCl layer on the surface of an Ag electrode (diameter of 3.0 mm, Metrohm, Switzerland) in a solution of 1 M HCl for 20 minutes at a constant current density of 1 mA cm^−2^.

#### Sodium ion-selective electrode

A glassy carbon (GC) electrode with a diameter of 3.0 mm purchased from Metrohm (Switzerland) was used as electrode substrate for solid-contact ion-selective electrode (ISE). Before use, it was polished with 0.3 μm alumina and washed with water. 5 layers (20 μL each) of single-walled carbon nanotubes functionalized with octadecylamine (SWCNT-ODA) (1 mg mL^−1^ in THF) were deposited onto the GC by drop-casting, each layer being allowed to dry for 10 min before the next deposition step. A PVC based ion-selective membrane for sodium determination was prepared by dissolving 100 mg of components in 1.0 mL THF: 15 mmol kg^−1^ of NaX ionophore, 5 mmol kg^−1^ of NaTFPB, 30 mmol kg^−1^ of ETH 500, 31.5 mg of PVC and 63 mg of DOS. It was then drop cast onto the SWCNT-GC electrode as follows: a volume of 150 μL (3 × 50 μL) was drop cast on the SWCNT-GC, each layer being allowed to dry 20 min before the next.

### Pulstrode protocol

The protocol consists of 4 steps: 1) open-circuit potential (OCP) measurement for 30 s. 2) Galvanostatic cathodic pulse of 5 s duration at either 5 μA (macro-electrode) or 4.5 μA (SPE and IPE). 3) Potentiometric measurement (EMF) for 0.25 s. 4) Potentiostatic pulse at an applied potential of the value recorded in step 1) for a period of 30 s.

### Preparation of artificial urine

The artificial urine solution was prepared by dissolving the following salts in 1 L of deionized water: 3.825 g of potassium chloride, 8.5 g of sodium chloride, 24.5 g of urea, 1.03 g of citric acid, 0.34 g of ascorbic acid, 1.18 g of monopotassium phosphate, 1.4 g of creatinine, 0.64 g of potassium hydroxide and 0.47 g of sodium bicarbonate. 0.28 mL of concentrated sulfuric acid were also added to the solution.

### Extenal potentiometric calibrations

The response of sodium- and chloride-selective electrodes to variations in sodium or chloride ion activity was evaluated using two types of reference electrodes, a conventional Ag/AgCl double-junction electrode and an inkjet-printed Ag/AgI reference electrode. Ion activity was progressively increased from 10^−4^ to 10^−1^ M by successive additions of sodium chloride solutions as follows. For each experiment, 50 mL of deionized water was placed in a glass beaker into which the reference and ion-selective electrodes were immersed and continuously stirred using a magnetic stirrer. Sodium chloride solutions were then added in the following sequence: 455 μL of 10^−2^ M NaCl, 459 μL of 10^−1^ M NaCl, 463 μL of 1 M NaCl, and finally 5.138 mL of 1 M NaCl.

### Single-point calibration method for urine quantification

The urine samples used were pooled urine provided by CSEM, which were treated as follows: 10 mL of urine is mixed with 250 mg of active carbon powder, centrifugated at 5000 rpm for 5 min and the supernatant was filtered through a PES filter.

The ISEs were measured against either the IPE-RE (working electrode 1) or a double junction Ag/AgCl reference electrode (Metrohm, Switzerland) (working electrode 2). A first potentiometric measurement in artificial urine was obtained, which allowed one to relate the potential of the respective configuration (ISE *vs.* IPE-RE and ISE *vs.* classical RE) to a precisely known concentration of the analyte of interest. Using the experimentally verified Nernst response of the ISEs, a subsequent potentiometric measurement in biological urine allowed one to determine the concentrations of chloride and sodium in that matrix.

## Results and discussion

**Fig. 3:**
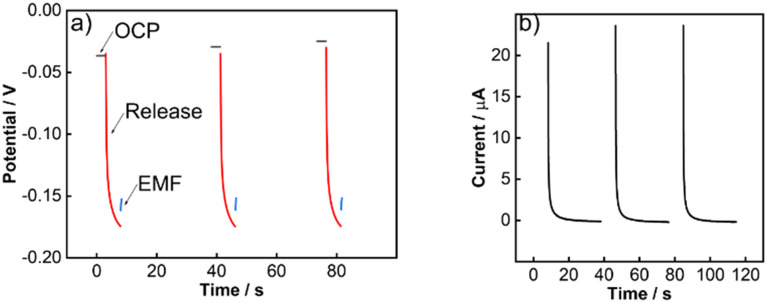
Experimental traces for the pulstrode protocol steps with a macro-electrode: (a) open-circuit measurement for 3 s (black trace), galvanostatic pulse at 5 μA for 5 s (red trace) and EMF measurement for 0.25 s (blue trace) and (b) potentiostatic (OCP + 50 mV) regeneration pulse for 30 s. Background electrolyte: 0.15 M NaCl.

The pulstrode protocol consists of four distinct steps: the measurement of the open-circuit potential, the release of iodide, the measurement of the consequent EMF at *t* = 0 s which serves as reference potential, and the regeneration of the silver iodide layer (Fig. 3a and b). In the first stage of this study, a macro-electrode was used to establish the behavior of a well-behaved silver/silver iodide system.

### Potentiometric response to iodide

The EMF of the Ag/AgI electrode is expected to depend on the activity of iodide *a*_I^−^_ at the electrode according to the Nernst equation (eqn (1)):

1 EMF = *E*^0^ − *s* log *a*_I^−^_

where *s* is the electrode slope (ideally 59.2 mV at 298 K). The theoretical value of *E*^0^ is −361 mV, consisting of the standard electrode potential *E*^0^_Ag/AgI_ minus the potential of the half-cell of the Ag/AgCl reference element.

### Open-circuit potential and lower detection limit

When approaching the lower limit of detection, the EMF response of the electrode starts deviating from the Nernst equation. Ideally, the EMF is dictated by the dissolution of the silver iodide salt, either defined by the solubility product *K*_s_(AgI) or by *α*, which is the silver defect concentration at the surface of the electrode.^38^

As a result, the behaviour of the electrode is described differently depending on the conditions.

For *a*_I^−^_ > *α*:2
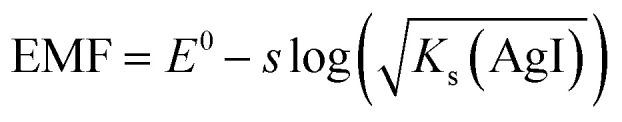


And for *a*_I^−^_ < *α*:

3 EMF = *E*^0^_Ag_ + *s* log(*α* − *a*_I^−^_)

The open circuit potential of an Ag/AgI electrode in the absence of iodide background is typically dominated by the silver defect ions α, estimated as 10^−6^ M.^38^

### Galvanostatic release of iodide

The quantity of iodide released at the electrode surface, [I^−^]_*d*=0_, is dependent on the applied current intensity, *i*_app_, and electrolysis time, *t*. It is ideally given by a relationship described by Bard and Faulkner for a chronopotentiometric experiment^39^ as follows:4
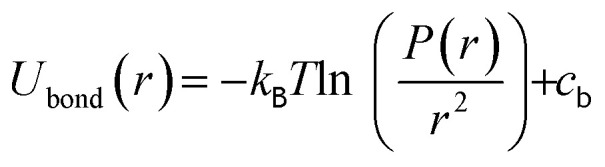
where *D*_aq_ is the diffusion coefficient of iodide in water, *F* the Faraday constant and *A* the geometric electrode area.

A geometrical, semi-empirical formulation of the release process may be helpful for understanding the principle. In the absence of iodide in the sample bulk, the concentration of iodide at the surface of the electrode may be formulated with Fick's first law. Expressing the one-dimensional flux as a function of diffusional current gives:5
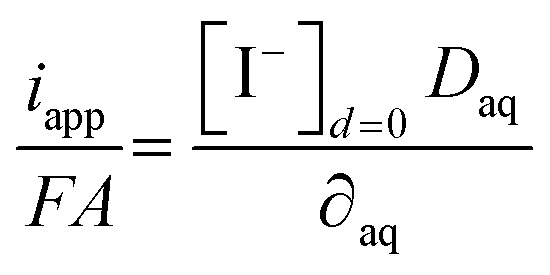
where ∂_aq_ is the diffusion layer thickness within which the iodide concentration is assumed to decrease linearly from [I^−^]_*d*=0_ to zero. From this assumption the number of moles of released iodide can be estimated as [I^−^]_*d*=0_*A*∂_aq_/2. This quantity is a function of the charge passed (*I*_app_*t*) and results in eqn (6):6
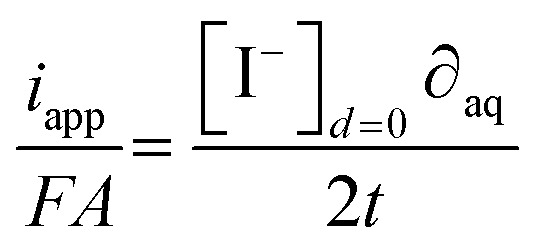
Setting both right sides of eqn (5) and (6) equal, the time-dependent aqueous diffusion layer thickness ∂_aq_ is found to depend on time as follows:^39^7
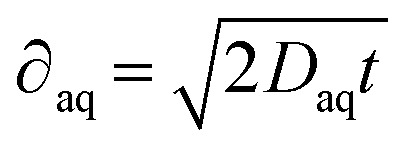
The concentration of iodide [I^−^]_*d*=0_ at the surface of the electrode at an electrolysis time *t* is then approximated as follows:8
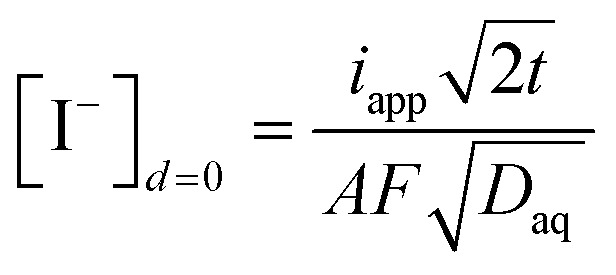
The assumption of a linear concentration gradient in the semi-empirical approach is not strictly correct, so the more correct analytical solution eqn (4) differs from eqn (8) by a factor of 
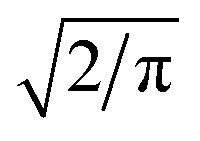
 = 0.80.

With a diffusion coefficient *D*_aq_ = 1.95 × 10^−7^ dm^2^ s^−1^, an applied current *i*_app_ = 5 μA and a geometric electrode area *A* = 0.0706 cm^2^, the potential change during the release pulse can be estimated by inserting the surface concentration into eqn (1) using concentration instead of activity. The predicted time trace based on eqn (4) (Fig. S1† – red line) correlates indeed better with the experimental data (Fig. S1† – blue line) than the predicted trace based on semi-empirical eqn (8) (Fig. S1† – grey line).

### EMF measurement step

The subsequent EMF value should correspond to the last data point of the release pulse. Excellent correlation between the predicted and experimental values of respectively −166.6 and −168.9 mV were obtained. However, this does not account for the ohmic drop which corresponds to a variation of the solution resistance, which leads to a potential shift. To eliminate this influence, the reference potential was sampled at zero current, giving an experimental EMF value at *t* = 0 of −158.1 mV during pulse 3. The response of the electrode to changes in iodide activity is shown in Fig. S2.† This allows one to correlate the EMF to the activity of iodide at the surface of the electrode at the end of the release pulse using the Nernst equation (eqn (1)). The experimental EMF value of −158.11 mV corresponds to an activity of 0.441 mM. In a background of 0.15 M KNO_3_, this EMF value corresponds to a surface concentration of 0.575 mM. This is in excellent agreement with the theoretical model described above that predicts a surface concentration of 0.525 mM.

### Regeneration step and interferences

The final step of the pulstrode protocol is a regeneration step. Upon application of an appropriate potentiostatic pulse, silver is again oxidized, resulting in the selective re-plating of iodide as silver iodide on the electrode. However, an excessive potential may result in the undesired plating of other solution ions such as chloride. Chloride is a ubiquitous ion in most samples and its co-plating must be avoided. The concentration of iodide at the surface of the electrode during the regeneration pulse, [I^−^]_lim_, is dictated by the applied potential *E*_regen_, written here for concentrations instead of activities:

9 *E*_regen_ = *E*^0^_Ag/AgI_ − *s* log[I^−^]_lim_

The ratio of the two solubility products relates the limiting concentration of iodide at the electrode surface to the anticipated chloride background before co-plating is observed:10
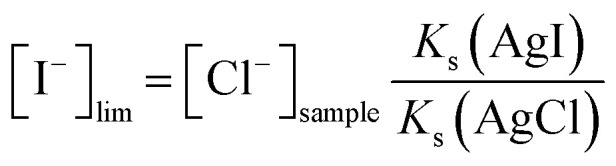
With *K*_s_(AgI) = 1.4 × 10^−16^ and *K*_s_(AgCl) = 1.2 × 10^−10^, the concentration of iodide during the regeneration step should be at least:

11 [I^−^]_lim_ = 1.2 × 10^−6^[Cl^−^]_sample_

Therefore, for a background chloride concentration of 0.1 M, the limiting iodide concentration at the electrode surface during regeneration is 0.1 μM (eqn (11)). With an OCP value (37 mV) before the release pulse, estimated to correspond to the detection limit of 1 μM, the applied potential during the regeneration pulse should not exceed OCP + 59 mV. However, the ohmic drop can distort the potential values and more conservative numbers should be used.

In the initial work presented by Gao *et al.*,^35^ the potential applied during the regeneration pulse was OCP + 50 mV. However, in the presence of an elevated sodium chloride background, co-plating of chloride was observed with time, resulting in a dramatic potential increase of around +200 mV after just 5 to 6 consecutive cycles (Fig. S3†). When the same parameter of OCP + 50 mV is applied in the presence of potassium nitrate background, or when the applied potential is only the initial OCP but in a sodium chloride background, no problematic shift of potential was observed, and the electrodes are stable over 25 cycles. We may conclude that it is important for the long-term stability of the electrode to only apply the initial OCP value during the regeneration step.

### Screen-printed electrodes (SPE)

The suitability of screen-printing for the Ag/AgI pulstrode protocol was evaluated. The Ag/AgI element was prepared by oxidizing the silver layer in the presence of iodide ions, resulting in the formation of a silver iodide layer. However, in the case of SPEs, the available silver layer is much more limited than with a macroelectrode, and excessive oxidation should be avoided. The quantity of silver was estimated by a coulometric experiment during which the silver is exhaustively converted to silver iodide (Fig. S4†). Coulometric experiments demonstrated a good inter-electrode reproducibility regarding the silver quantity, with an average observed charge of 10.6 ± 1.7 mC (standard deviation, *N* = 3). Based on the dimensions of the electroactive area and the known density of silver, the silver layer is estimated to be of 358 ± 56 nm thick.

This experiment provides the important information on the quantity of silver to be converted into silver iodide to maintain an adequate conductive layer of silver underneath the silver iodide. A fraction of 30% of the available silver was converted into silver iodide. This deposition occurred on the time scale of minutes. The functionality of the prepared electrodes was verified by measuring the electrode response to changes in iodide activity (Fig. S5†). Quasi-Nernstian slopes of −56.2, −53.1 and −46.2 mV respectively were obtained potentiometrically in the concentration range of 0.1 mM to 100 mM using the Ag/AgI electrodes and a conventional Ag/AgCl-based reference electrode with liquid junction.

The pulstrode protocol was applied to screen-printed electrodes (Fig. S6a and b†), and the stability over 25 cycles in potassium nitrate background was assessed for electrodes prepared in the aforementioned manner (Fig. S7†). The EMF values for electrode 2 differed importantly from the EMF values of electrode 1 and 3. This abnormal behavior may be attributed to the surface area of the electrode differing from the others, resulting in an altered current density being applied during the release pulse. Variation of the EMF values of between the first and the last cycle of −4.6, −2.6 and −6.3 mV for electrode 1, 2 and 3 were observed, respectively. The drift of the electrodes is thought to be caused by the many impurities present in the silver ink. Based on the electrochemical data as well as the inherent characteristics of screen-printing, *i.e.* the difficulty to obtain a very reproducible surface area of the electrode and the ink impurity, it was concluded that screen-printing was not an adequate process for the fabrication of Ag/AgI pulstrode reference elements with the available silver ink. The visual observation of a screen-printed electrode before deposition (Fig. S8a†), after deposition (Fig. S8b†) and after 25 cycles of pulstrode protocol (Fig. S8c†) demonstrates the undesired changes in the material with time.

### Inkjet-printed electrodes (IPE)

The suitability of inkjet-printing to manufacture the substrate for the Ag/AgI pulstrode protocol was evaluated. Coulometric experiments (Fig. S9†) demonstrated a good inter-electrode reproducibility regarding the silver layer quantity, with an average charge of 10.8 ± 0.3 mC (standard deviation, *N* = 3). Based on the dimension of the electro-active area and the density of silver, the silver layer was estimated to be 365 ± 10 nm thick (standard deviation) from this charge.

**Fig. 4:**
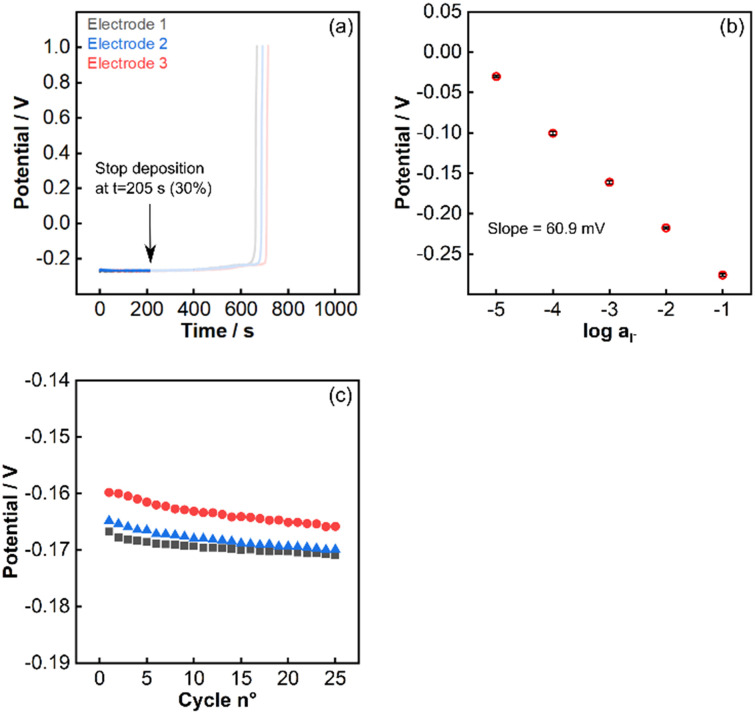
Potentiometric traces for the deposition of the silver iodide layer, corresponding to a conversion of 30% of the silver layer (a). Response to changes in iodide activity (b) and reference values produced by the pulstrode protocol (c) of electrodes with 30% conversion.

Various fractions of silver were converted to silver iodide (10, 30, 60 and 90%). The functionality of each ratio of silver iodide to silver was verified by measuring the electrode response to changes in iodide concentration (Fig. S10a–d†). Nernstian slopes of −60.2, −60.9, −60.9 and −61.8 mV were obtained, respectively. The pulstrode protocol was applied to the inkjet-printed electrodes (Fig. S11a and b†), and the stability of the pulstrode protocol was assessed over 25 cycles (Fig. S12a–d†). A conversion of 10% resulted in one of the three electrodes to exhibit potentials 250 mV lower than the others during the pulstrode protocol. This was attributed to an incomplete coverage of the silver by the silver iodide layer. On the other hand, a conversion of 90% resulted in unstable EMF values during the pulstrode protocol while the conversion of 60% resulted in attractive potential stability for only two electrodes. The abnormal behavior of electrode 2 was attributed to a manufacturing issue within the inkjet-printing step. Only a conversion of 30% resulted in reasonably reproducible inter-electrode EMF measurements, with drifts of −4.1, −6.0 and −5.1 mV over 25 cycles of pulstrode protocol, respectively. The ratio of 30% silver iodide to silver was selected for further experiments (Fig. 4). However, none of the electrodes demonstrated an EMF stability over 25 cycles sufficiently satisfying to act as a reference element. The visual observation of an inkjet-printed electrode before deposition (Fig. S13a†), after deposition (Fig. S13b†) and after 25 cycles of pulstrode protocol (Fig. S13c†) resulted in no obvious visual differences.

The effect of the amplitude of the release pulse on the stability and reproducibility of the reference pulse was investigated (Fig. S14†) Variable current amplitude of 1, 3, 4.5, 7 and 10 μA were applied for 10 consecutive cycles on three different electrodes. Average drifts of respectively −4.8, 2.4, −1.1, −1.6 and −5.0 mV were obtained. For electrodes of 2 mm diameter, a current pulse of −4.5 μA for 5 s was adopted. However, observing the overall trend of electrode 1 compared to electrode 2 and 3 highlights that while the amplitude of the release pulse can impact the stability of the EMF values over cycles, the intrinsic characteristic (emerging from the inkjet-printing manufacturing step) determines the quality of the electrode and its consequent suitability for the pulstrode protocol.

**Fig. 5:**
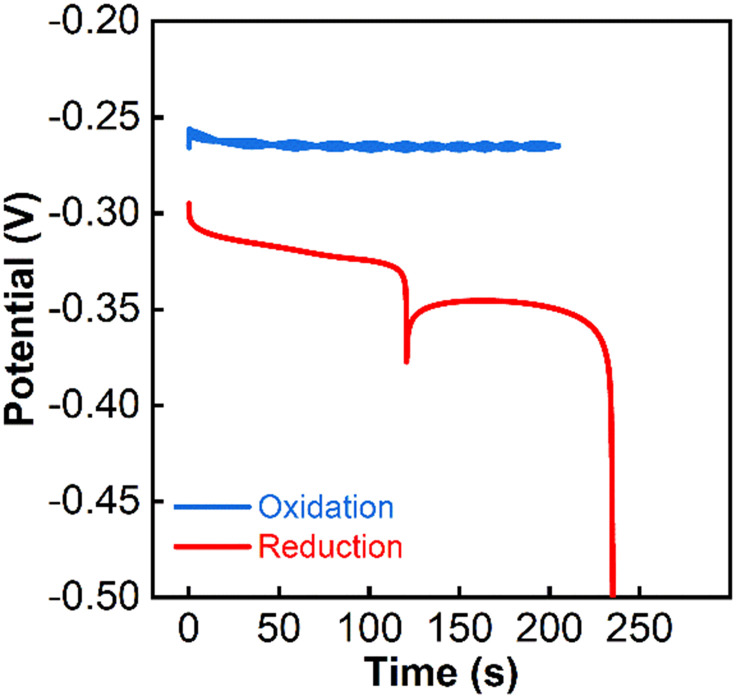
Potentiometric traces under application of galvanostatic currents of 15.7 (blue) and −15.7 μA (red) in 0.1 M NaI solution for one inkjet-printed electrode.

Other electro-active species interfering with the pulstrode protocol may be present on the electrode, likely as impurities present in the ink. An anodic current pulse in sodium iodide, which should result exclusively in the conversion of silver to silver iodide, and a subsequent cathodic current pulse in an inert salt solution (potassium nitrate) was performed. It resulted in a discrepancy of the observed charges (respectively 3.2 (anodic pulse) and 3.9 mC (cathodic pulse)) (Fig. 5). The sudden potential drop at about 250 s during the cathodic pulse is associated with the depletion of all electro-active species on the electrode. The charge mismatch between cathodic and anodic pulse indicates the presence of already oxidized impurities, which might be interfering with the pulstrode protocol. The spike at around 100 s during the cathodic pulse correlates with the time at which the area of the electrode visibly starts to diminish (Fig. S15†). The procedure was repeated for three electrodes in order to make sure the result was not an aberration (Fig. S16a–f†).

Energy Dispersive X-ray spectroscopy (EDX) experiments provided information about the atomic composition of inkjet-printed electrodes (Table S1†). The presence of roughly 5% of impurities, mostly silicon and aluminum, can be noted on the bare silver electrode (Fig. S17†). The suggested presence of tellurium in the sample is thought to be due to traces in the stainless steel of the instrument. The electrodes deposited with silver iodide still contained impurities, and whether the electrode deposition was new (Fig. S18†) or used (Fig. S19†) did not impact its composition in a significant manner.

**Fig. 6:**
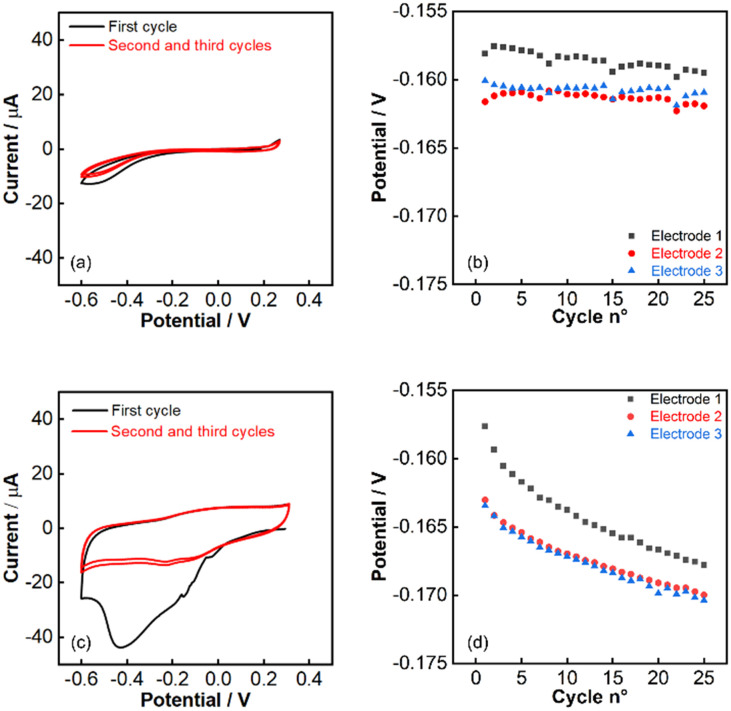
Cyclic voltammetry experiment of bare silver macro-electrode (a) and inkjet-printed electrode (c) in 0.15 M potassium nitrate solution. 25 cycles of pulstrode protocol in 0.15 M potassium nitrate after electrochemical cleaning on (b) macro-electrode and (d) inkjet-printed electrode (3 cycles, 50 mV s^−1^, from −0.6 to 0.3 V).

The comparison of cyclic voltammetry experiments performed on the macroelectrode and the inkjet-printed electrode in potassium nitrate confirmed the presence of impurities in the ink (Fig. 6a and c). Much of the electro-active impurities could be removed with a single cycle. This suggested that a pre-deposition cleaning step using cyclic voltammetry may improve the stability of the pulstrode protocol. Drifts of −10.1, −7.0 and −6.9 mV were obtained respectively (Fig. 6d) for electrodes having undergone an electrochemical cleaning step using cyclic voltammetry prior to the silver iodide deposition step. Electrochemical cleaning did not change significantly the stability of the pulstrode protocol for the macroelectrode (Fig. 6b).

Visual observation of five different electrodes after deposition demonstrates a variability in the electroactive area (Fig. S20a–e†). Quantifying the electroactive area *versus* the global surface area shows that only 71.4 ± 4.0% (standard deviation, *N* = 5) of the electrode surface is actually accessible for silver iodide deposition. The microscope imaging shows this variability is the result of a leakage of the dielectric onto the silver layer (Fig. S21a and b†). Consequently, the silver covered by dielectric cannot be converted into silver iodide during the electrochemical conversion step. This variability in electro-active area is thought to be largely responsible for the inter-electrode variability in EMF. With a constant current amplitude, the released iodide concentration is inversely proportional to the electrode area (see *A* in eqn (4)).

To investigate this hypothesis, the electro-active area of two electrodes (Fig. S22a and b†) was quantified (Table S2†) and used to predict the potentials during the release pulse (Fig. S22c†). The difference between the predicted values when correcting the current density for the area indeed resulted in a compression of the data (Fig. S22d†), suggesting that the variability of the electro-active area is indeed responsible for the initial inter-electrode EMF variability. Electrochemical techniques such as cyclic voltammetry may provide information about the geometric electro-active area of an electrode so that the applied current density during the pulstrode protocol can be adjusted. However, these techniques, as well as visual quantification, imply an extra step and therefore a longer and more complicated fabrication process of the IPE. On the other hand, electrochemical impedance spectroscopy gives area information at the microscopic level, which is less relevant here.

Another important aspect is the stability of the deposited electrodes over time. It was observed that electrodes deposited and stored in the dark for 24 hours could exhibit different EMF values after one cycle of pulstrode protocol (Fig. S23†). Electrodes stored under dark and light conditions (Fig. S24a and b†) showed an unpredictable first EMF value, while electrodes freshly deposited did not (Fig. S24c†). This issue can effectively be addressed by excluding the first pulse. The average drifts after 25 cycles of pulstrode electrodes after being stored in the dark, stored in the light and freshly deposited were −2.8, 5.9 and −5.1 mV, respectively.

Potentiometric traces of the release and reference pulses of two electrodes sequentially deposited with iodide and stored under the same conditions were recorded (Fig. S25a–g†). Other electroactive compounds appear to be present on the electrodes and in variable quantities. The application of a galvanostatic cathodic pulse after the deposition and at the start of the pulstrode protocol may improve the reproducibility as well as the stability of the EMF values.

Despite electrochemical, spectroscopic, and microscopic data all being consensual regarding the presence of impurities, it was difficult to completely remove this influence, which calls for an obvious need to optimize the manufacturing process. Despite this, the suitability of the inkjet-printed electrode for quantification of major ions in urine was explored.

The response of chloride- and sodium-selective electrodes in aqueous solution against a conventional reference electrode as well as against the inkjet-printed electrodes was investigated. For chloride, the conventional reference electrode and the inkjet-printed reference element (IPE) provided slopes of −57.6 and −52.1 mV respectively. For sodium, slopes of 57.1 and 55.6 mV were obtained with the conventional reference electrode and the IPE respectively (Fig. S26a–d†).

Urine samples, provided by CSEM and analyzed by an external laboratory, were treated in accordance with established protocols for clinical analysis (see Exp. sec.) and used to verify the suitability of both reference elements for urine analysis. Using potentiometric external calibrations, both reference elements allowed to quantify chloride and sodium in urine within a satisfying margin of error relative to the values provided by the reference analysis (27 mM for both chloride and sodium) with values of 28.7 mM (sodium – conventional RE), 31.7 mM (sodium – IPE), 25.8 mM (chloride – IPE) and 22 mM (chloride – conventional RE). It can already be noted that the value found with the IPE for chloride is closer to the reference value than for sodium, the reasons for which are discussed below.

**Fig. 7:**
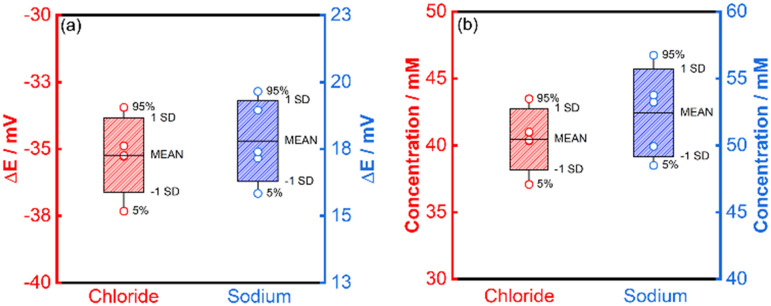
Potentiometric quantification of urine ions using inkjet-printed electrodes (*N* = 5) (a) potential differences between synthetic (calibrant) and biological urine and (b) concentration measured in biological urine.

A single-point calibration method was adopted for the determination of chloride and sodium in activated carbon-filtered urine (Fig. S27†). Five different inkjet-printed electrodes were used for each ion. The quantification of chloride showed excellent agreement between the results obtained for the IPEs, the conventional RE and IC (Fig. S28a and b†), with values of 40.5, 43.8 and 39.5 mM respectively (Table 1 and Fig. 7a and b). The quantification of sodium, while still deemed successful, showed a lesser agreement between the IPEs, conventional RE and AES, with values of 52.4, 46.0 and 40.2 mM respectively.

**Table 1:** Summary of the measurement resulting from single-point calibrations for inkjet-printed electrode and double-junction silver/silver chloride reference electrode against chloride and sodium ion-selective electrodes, and comparison of the values obtained by Ion Chromatography (IC) or Atomic Emission Spectroscopy (AES)

	Classical reference electrode (mM)	Average inkjet-printed (mM)	IC/AES (mM)
Chloride	43.8	40.5 ± 2.28	39.5
Sodium	46.0	52.4 ± 3.26	40.2

This difference in agreement between the conventional reference electrode and the inkjet-printed electrodes for chloride and sodium originates in the charge of the two analytes. The reference potential of the inkjet-printed electrode is dictated by the iodide ions in the vicinity of the electrode. From eqn (12) and (13), the potential of the cell is affected differently by the activity coefficients whether the measured ion is cationic or anionic. The overall cell potential for the detection of sodium is written as:12
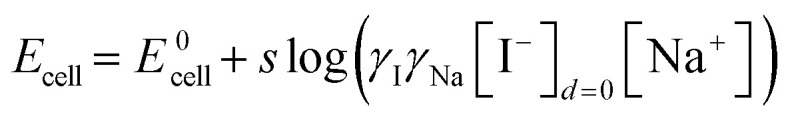
While for measuring chloride:13
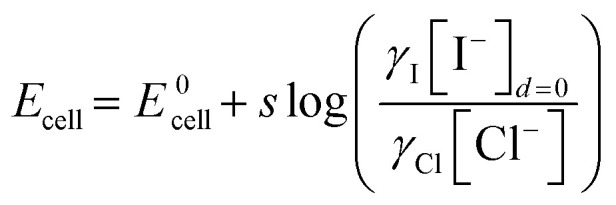
In the case of chloride, the activity coefficients may be assumed to be similar to iodide, and the potentiometric reading can be directly correlated to the concentration of chloride. In the case of sodium, however, the activity coefficient should be considered to correlate the potentiometric reading and concentration. The activity coefficient itself depends on the ionic strength, which in turn is dictated by the total content of all charged species in a medium. Urine samples exhibit significant variability of ionic strength. Therefore, an estimated activity coefficient was used for the calculations involved. For these reasons, the pulstrode utilizing an Ag/AgI element is more reliable for the detection of anions than for cations.

The average relative error of 7.7% for (chloride) of the inkjet-printed electrodes *versus* the conventional RE (Fig. S29†) is still within the margin of error generally accepted for medical applications of other metabolites,^40^ while the value of 14.1% (sodium) is marginally above the permissible limit.

## Conclusion

Commercially available silver inks were used to screen-print and inkjet-print a silver layer on top of a polyethylene substrate. Using screen-printing, a dielectric was used to define the electro-active area. Subsequently, a fraction of this layer was converted by controlled current electrolysis into silver iodide, yielding a functional silver/silver iodide electrode. The application of a pulstrode protocol allowed one to use these as reference elements that self-generate their potential. The quality of the manufactured reference elements was assessed by potentiometry. Screen-printed reference electrodes showed to be inadequate, as the reference potential was not stable over time. This drift was assumed to result from the low purity of the silver ink, which contains binders and additives in considerable quantities. Inkjet-printed electrodes, manufactured using ink with a high degree of silver content, exhibited better performance than the screen-printed electrodes. However, the lack of *E*^0^ inter-electrode reproducibility and the still considerable drift over time made it not yet suitable for long term applications. A single-point calibration method was therefore used to quantify chloride and sodium in urine. The ISE responses were measured against the inkjet-printed electrodes as well as against a conventional silver/silver chloride electrode to compare. The electrochemical results were also cross-correlated using ion chromatography and atomic emission spectroscopy. The inkjet-printed electrodes provided a reliable reference potential that allowed to quantify the ions in urine within an acceptable margin of error. Intra and inter-batch variations is a remaining challenge of inkjet-printing, and it is evident that the manufacturing process, as well as the ink formulation still requires optimization to yield a stable and reproducible potential for long term applications without a pre-calibration step. Furthermore, the application of a hydrogel layer on top of the silver/silver iodide layer is expected to improve the stability and reproducibility of the reference pulse by protecting the released iodide from convection as well as slowing its diffusion between the release and regeneration steps. Improving these aspects is currently in progress in our laboratories.

## Compliance with ethical standards

The samples have been collected directly from volunteers working at CSEM SA – Center Landquart, and informed consent was obtained from all human subjects. All urine samples used in this research project have been collected and stored anonymously, without any type of list of donors or correspondence table. As this research therefore involves anonymised biological material, it falls out of the scope of the Swiss Federal Act on Research involving Human Beings (article 2) and thus does not require to be submitted to an ethics committee.

## Author contributions

Ayian Speck: conceptualization, methodology, investigation, software, data analysis, writing – original draft. Tara Forrest: methodology. Elena Zdrachek: conceptualization, methodology. Davide Migliorelli: resources. Guillaume Bouilly: resources. Jeremy Disser: methodology, investigation. Silvia Generelli: resources. Loïc Burr: resources, review. Eric Bakker: conceptualization, methodology, software, supervision, writing – review & editing.

## Conflicts of interest

The authors declare that they have no known competing financial interests or personal relationships that could have appeared to influence the work reported in this paper.

## Supplementary Material

SD-004-D5SD00024F-s001

## Data Availability

Original data for this work will be made available by the authors by reasonable request.
