# Supplementary material for: Microfabricated self-referencing pulstrodes
Source: Sens Diagn. 2025 May 14;4(8):669–79. doi: 10.1039/d5sd00024f (PMC12132089; doi:10.1039/d5sd00024f)
Supplement: SD-004-D5SD00024F-s001 [file SD-004-D5SD00024F-s001.pdf]

Supplementary Information (SI) for Sensors & Diagnostics.
This journal is © The Royal Society of Chemistry 2025

Supplementary Information forofabricated Self-Referencing Pulstrodes

Ayian Speck[1], Davide Migliorelli[2], Silvia Generelli[2], Guillaume Bouilly[2], Tara Forrest[1], Elena Zdrachek[1], Loïc Burr[2*], Eric Bakker[1*]

[1] Department of Inorganic and Analytical Chemistry, University of Geneva, Quai E.-Ansermet 30, 1211 Geneva 4, Switzerland
[2] CSEM Landquart, Bahnhofstrasse 1, 7302 Landquart, Switzerland

Table of Contents

Figure S1. Experimental and predicted release pulse. 2
Figure S2. Macro-electrode - response to iodide activity 3
Figure S3. Optimization of the regenration step. 3
Figure S4. Silver layer quantification by coulometry for screen-printed electrode 4
Figure S5. Observed potentiometric response at zero current of Ag/AgI screen-printed electrode to different activities of iodide. 4
Figure S6. Pulstrode protocol on screen-printed electrodes 5
Figure S7. Stability of Pulstrode protocol on screen-printed electrodes (25 cycles) 5
Figure S8. Photographs of a screen-printed electrode at different stages. 5
Figure S9. Silver layer quantification by coulometry for inkjet-printed electrodes. 6
Figure S10. Observed potentiometric response at zero current of Ag/AgI inkjet-printed electrode 7
Figure S11. Pulstrode protocol on inkjet-printed electrodes 8
Figure S12. Stability of pulstrode protocol on inkjet-printed electrodes following different conversion fraction of the silver layer. 8
Figure S13. Photographs of an inkjet-printed electrode at different stages. 9
FIGURE S14. DEPENDENCE OF THE REFERENCE PULSE STABILITY ON RELEASE PULSE AMPLITUDE. 9
Figure S15. Silver iodide layer reduction for inkjet-printed electrodes. 10
Figure S16. Silver layer oxidation and reduction for inkjet-printed electrodes. 10
Figure S17. Energy Dispersive X-ray spectrum of undeposited inkjet-printed electrode. 11
Figure S18. Energy Dispersive X-ray spectrum of freshly deposited inkjet-printed electrode. 11
Figure S19. Energy Dispersive X-ray spectrum of deposited (24hours) inkjet-printed electrode 12
Figure S20. Imaging of the variability in electro-active area. 12
Figure S21. Microscopic imaging of dielectic leakage.. 13
Figure S22. Release pulse and electro-active area correlation 13
Figure S23. Reproducibility of the first reference pulse after storage for 24hours for inkjet-printed electrodes 14
Figure S24. Stability and reproducibitliy of the reference pulse after different storage conditions. 14
Figure S25. Potentiometric observation of interfering electro-active species. 15
Figure S26. Observed potentiometric response to different activities of urine ions. 15
Figure S27. Schematic of the single-point calibration experimental set-up for urine ions quantificaiton 16
Figure S28. Ion-Chromatography and Atomic Emission Spectroscopy calibration curves 16
Figure S29. Relative errors of inkjet-printed electrode versus classical reference electrode in urine 17

Equations

The relationship between activity and concentration can be written as:

$$a_{I^-} = \gamma_{I^-} * [I^-] \qquad \text{Eq. S1}$$

The activity coefficient for iodide γ_{I^-} can be calculated using a semi-empirical relationship based on the extended Debye-Hückel theory (Eq. S2 and S3)

$$\log \gamma_{\pm} = -\frac{A|z_+ z_-|\sqrt{I}}{1 + B\sqrt{I}} + CI \qquad \text{Eq. S2}$$

The ionic strength of the solution I can be calculated with Eq. S3:

$$I = 0.5 \sum_j z_j^2 C_j \qquad \text{Eq. S3}$$

With A = 0.5108, B = 1.4651, C = 0.07233, z_j the charge of the ion j and C_j the concentration of ion j.

Figures

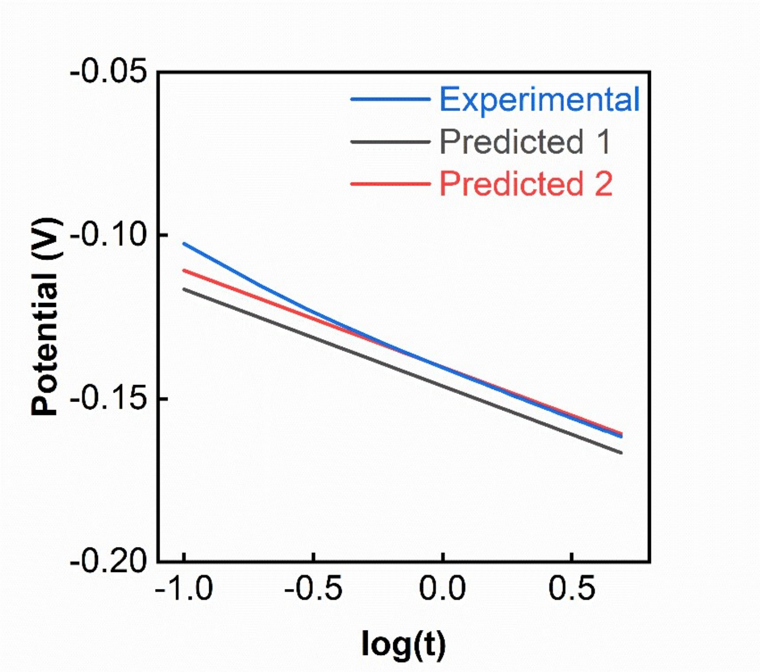

Figure S1. Experimental and predicted potentiometric traces upon application of a current amplitude = 5 μA for 5 s. The first model (grey trace) is based on geometric considerations, while the second model (red trace) is based on diffusion equations.

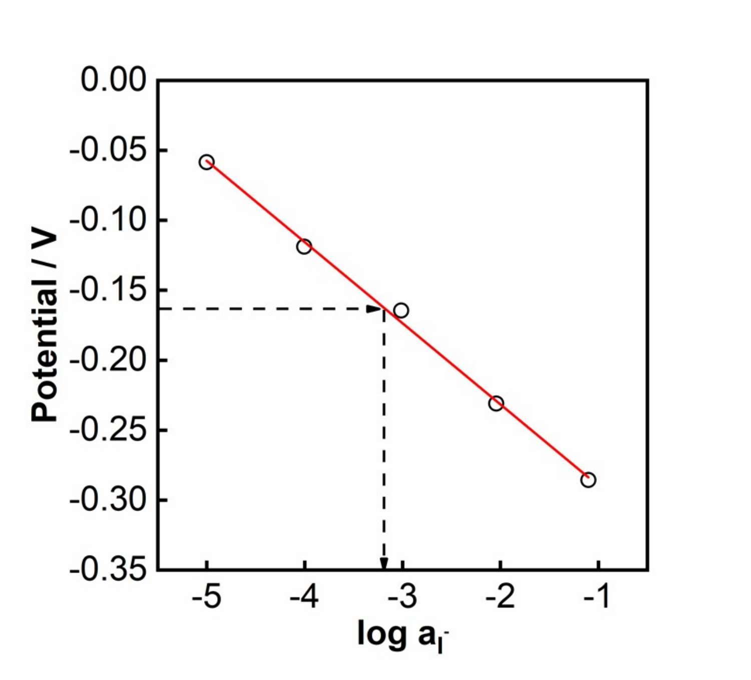

Figure S2. Observed potentiometric response at zero current of Ag/AgI macro-electrode to different activities of iodide ions in a background of 0.15 M KNO_3. Nernstian slope of 58.0 mV was obtained.

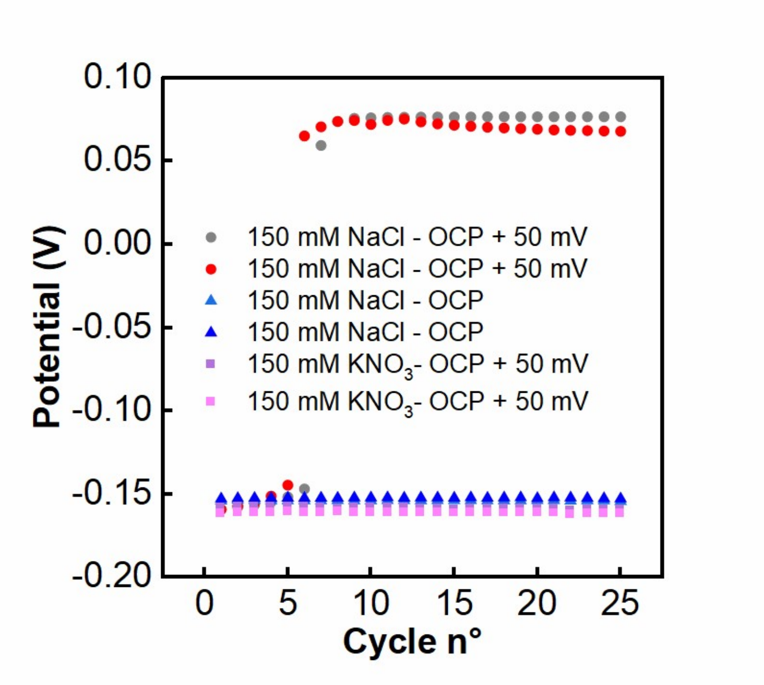

Figure S3. Stability of the reference pulses for 25 consecutive cycles of pulstrode protocol using a macro-electrode in different conditions. Parameters of the three steps (I) application of a current amplitude -5 µA for 5 s, (II) detection for 0.25 s, (III) application of a regeneration potential for 30 s.

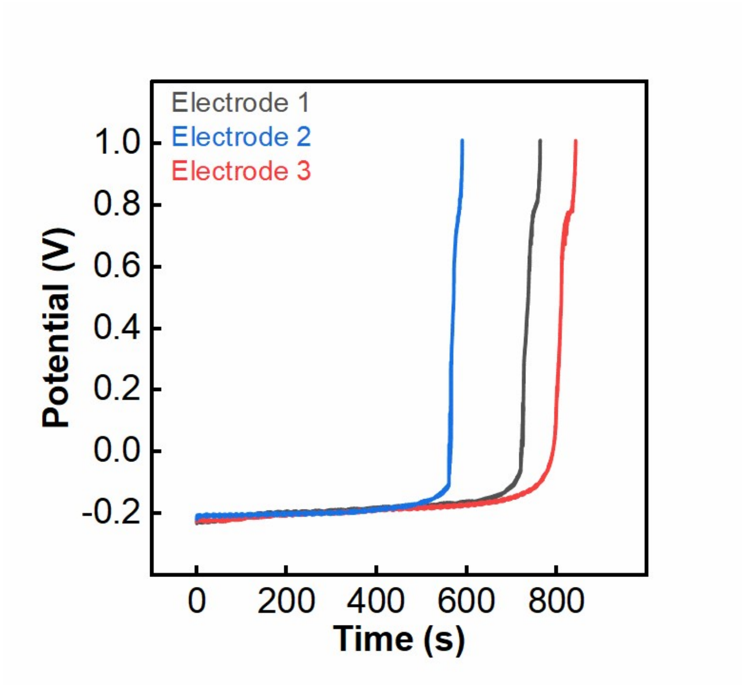

Figure S4. Coulometric experiment for three different screen-printed electrodes to quantify the accessible amount of silver in the layer. Reference element: Silver/silver chloride double junction and platinum counter electrode. Current applied: 15.7 µA. Background electrolyte: 0.1 M NaI.

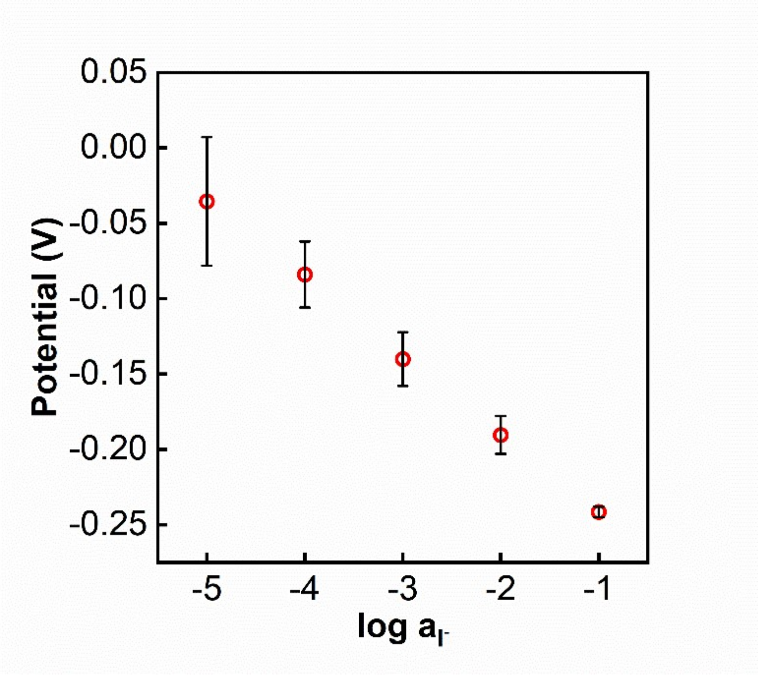

Figure S5. Observed potentiometric response at zero current of Ag/AgI screen-printed electrode to different activities of iodide ions in a background of 0.15 M KNO_3. Quasi-Nernstian slopes of -56.2, -53.1 and -46.2 mV were obtained respectively.

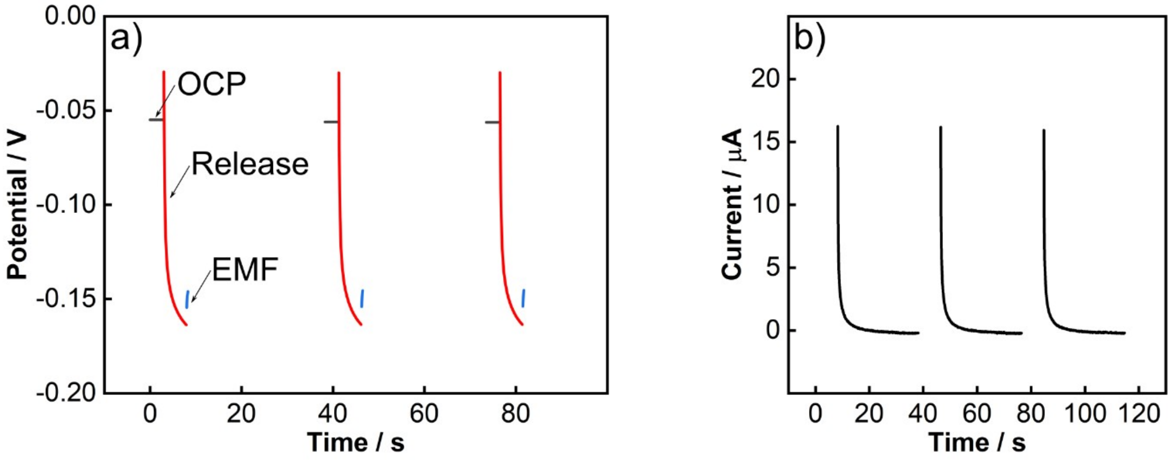

Figure S6. Experimental traces for the pulstrode protocol steps with a Screen-Printed Electrode: (a) Open-Circuit measurement for 3 s, galvanostatic pulse at -4.5 μA for 5 s and EMF measurement for 0.25 s and (b) potentiostatic (OCP + 50 mV) regeneration pulse for 30 s. Background electrolyte: 0.15 M NaCl.

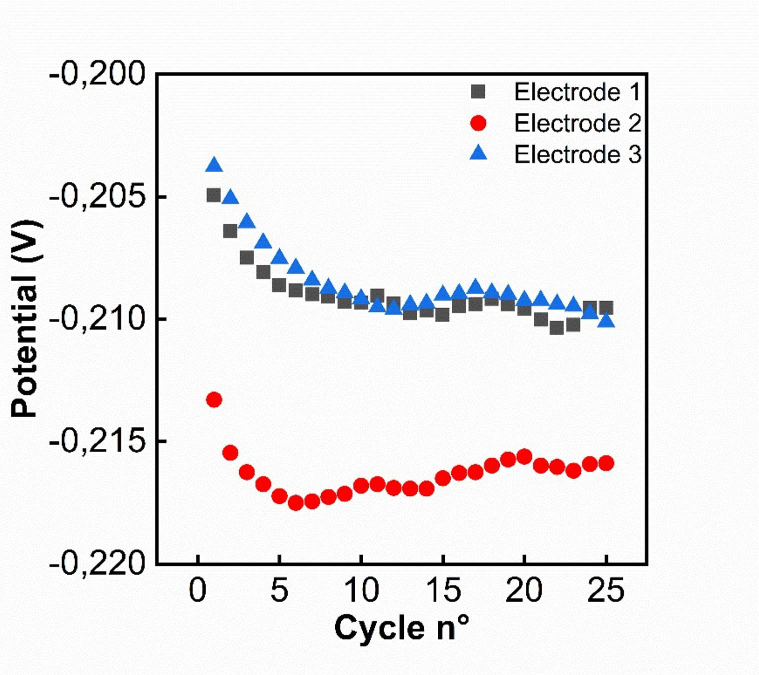

Figure S7. Stability of the reference pulses for 25 consecutive cycles of pulstrode protocol using screen-printed electrodes. Parameters of the three steps (I) application of a current amplitude -4.5 μA for 5 s, (II) detection for 0.25 s, (III) application of a regeneration potential equal to the initial open-circuit potential (OCP) for 30 s. Background electrolyte: 0.15 M KNO_3.

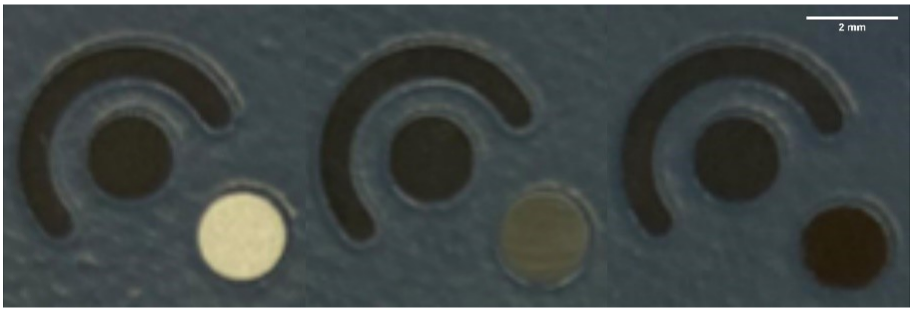

Figure S8. Photographs of a screen-printed electrode at different stages; undeposited (a), after deposition at 15.7 μA for 205 s (b) and after 25 cycles of pulstrode protocol (c).

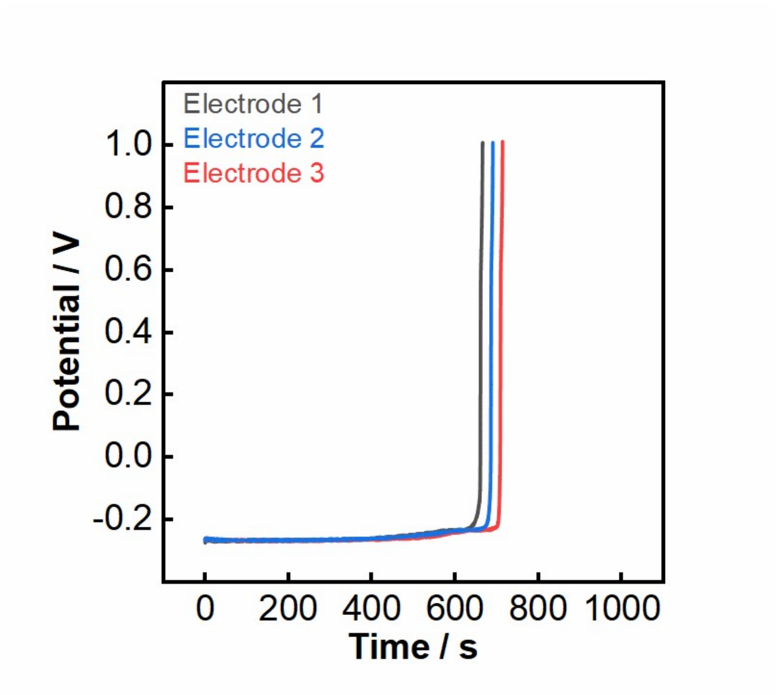

Figure S9. Coulometric experiment for three different inkjet-printed electrodes to quantify the accessible amount of silver in the layer. Reference electrode: Silver/silver chloride double junction and counter electrode: Platinum. Current applied: 15.7 μA. Background electrolyte: 0.1 M NaI.

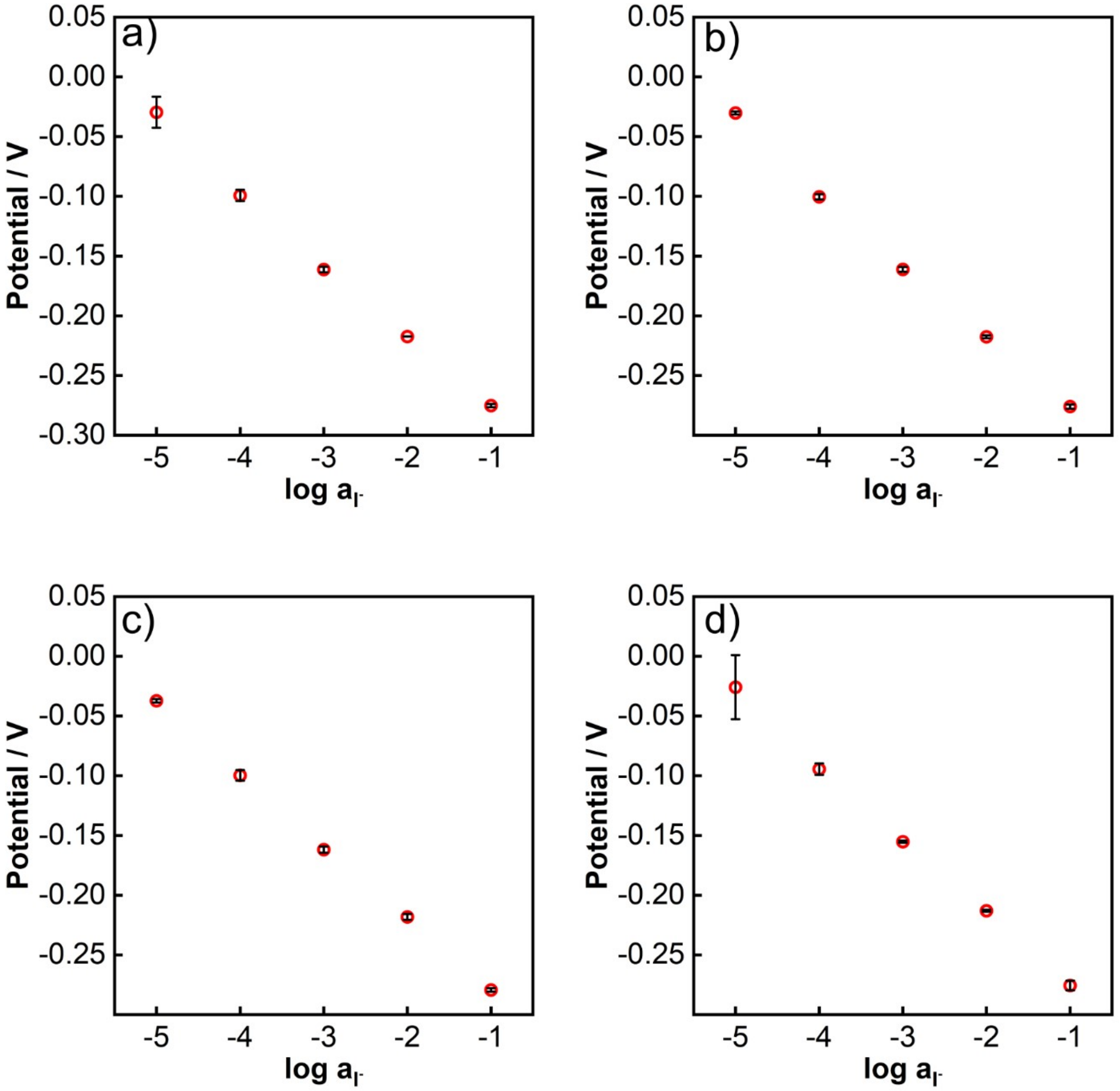

Figure S10. Observed potentiometric response at zero current of Ag/AgI inkjet-printed electrodes deposited for variable times to different activities of iodide ions in a background of 0.15 M KNO_3. Reference electrode: Silver/silver chloride double junction and counter electrode: Platinum. Current amplitude used for AgI deposition: 15.7 μA. deposition times: (a) 70 s, (b) 205 s, (c) 410 s and (d) 615 s.

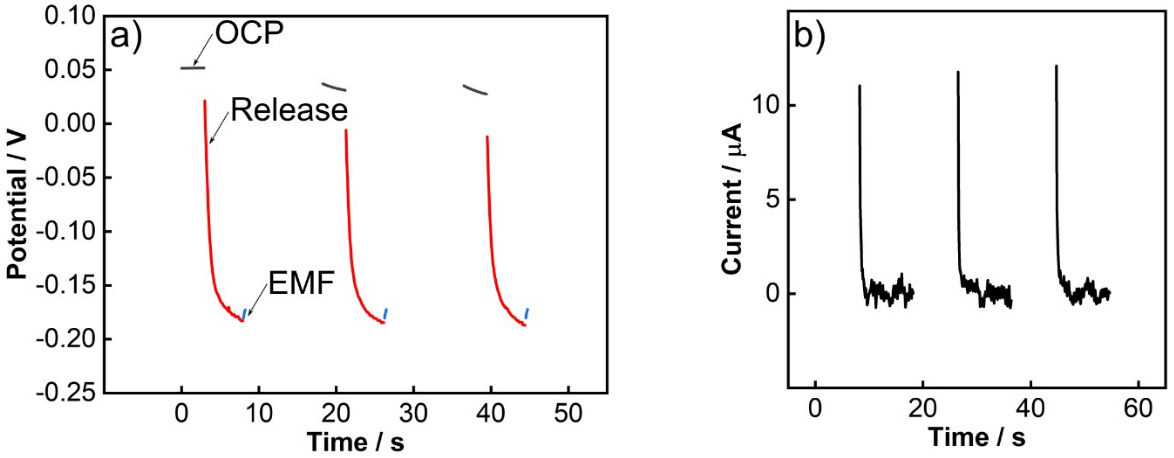

Figure S11. Experimental traces for the pulstrode protocol steps with an inkjet-printed electrode: (a) Open-Circuit measurement for 3 s, galvanostatic pulse at -4.5 μA for 5 s and EMF measurement for 0.25 s and (b) potentiostatic (OCP + 50 mV) regeneration pulse for 10 s. Background electrolyte: 0.15 M NaCl.

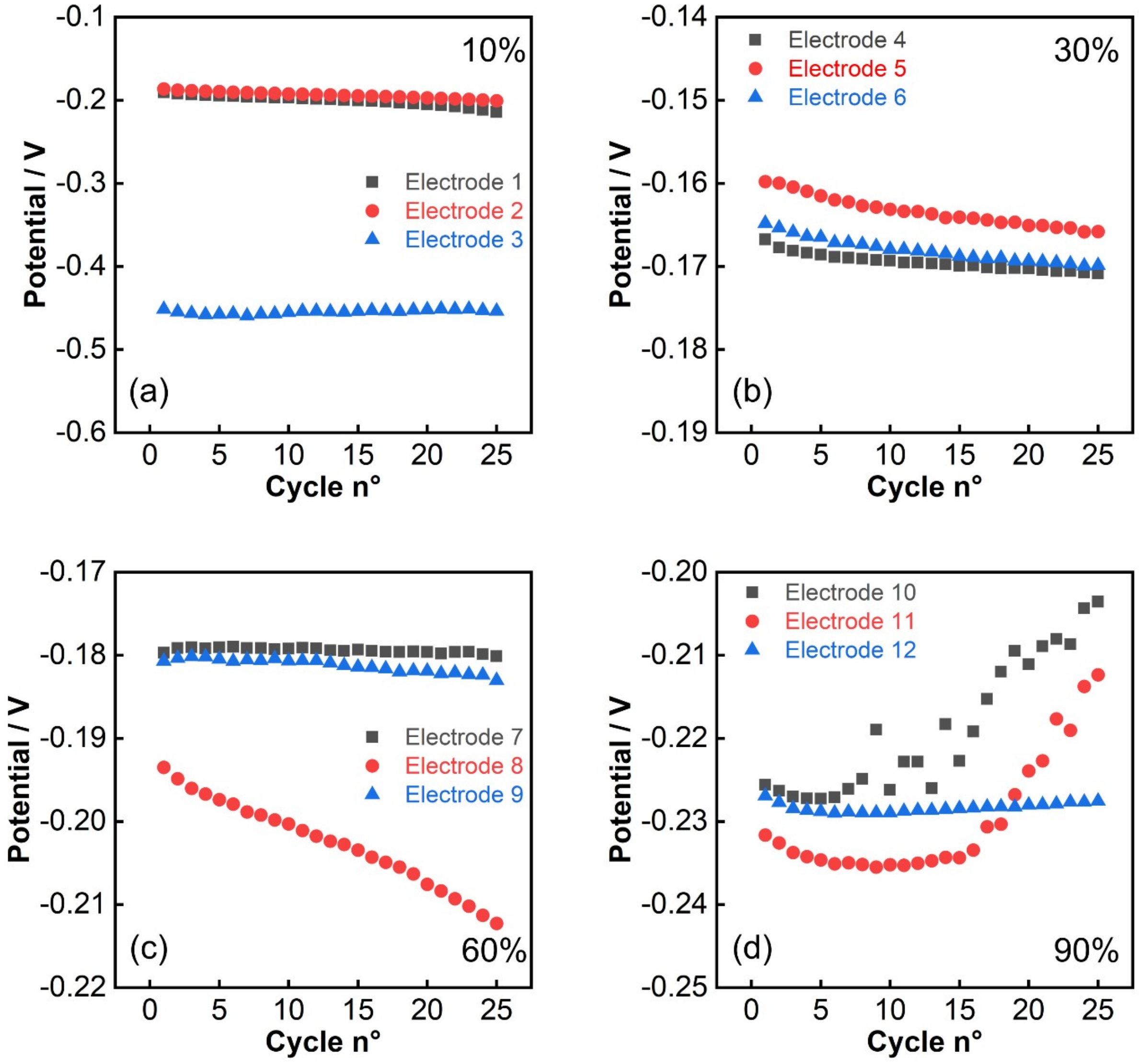

Figure S12. 25 cycles of pulstrode protocols in 0.15 M potassium nitrate for different fraction of silver converted into silver iodide (N=3) (current amplitude applied in prior deposition step: 15.7 μa. (a) 10%, (b) 30%, (c) 60% and (d) 90%.

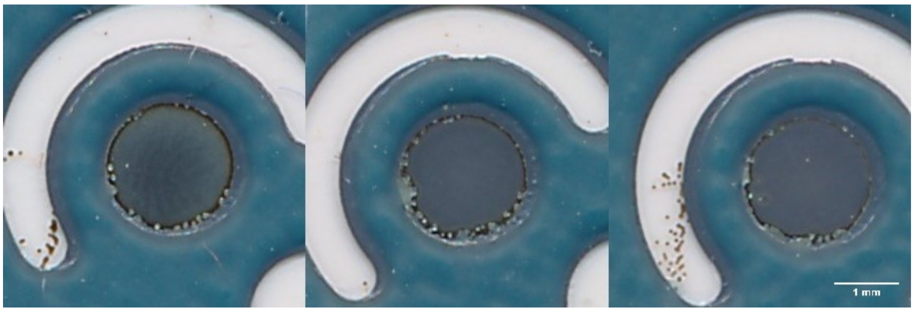

Figure S13. Photographs of an inkjet-printed electrode at different stages; undeposited (a), after deposition at 15.7 µA for 205 s (b) and after 25 cycles of pulstrode protocol (c).

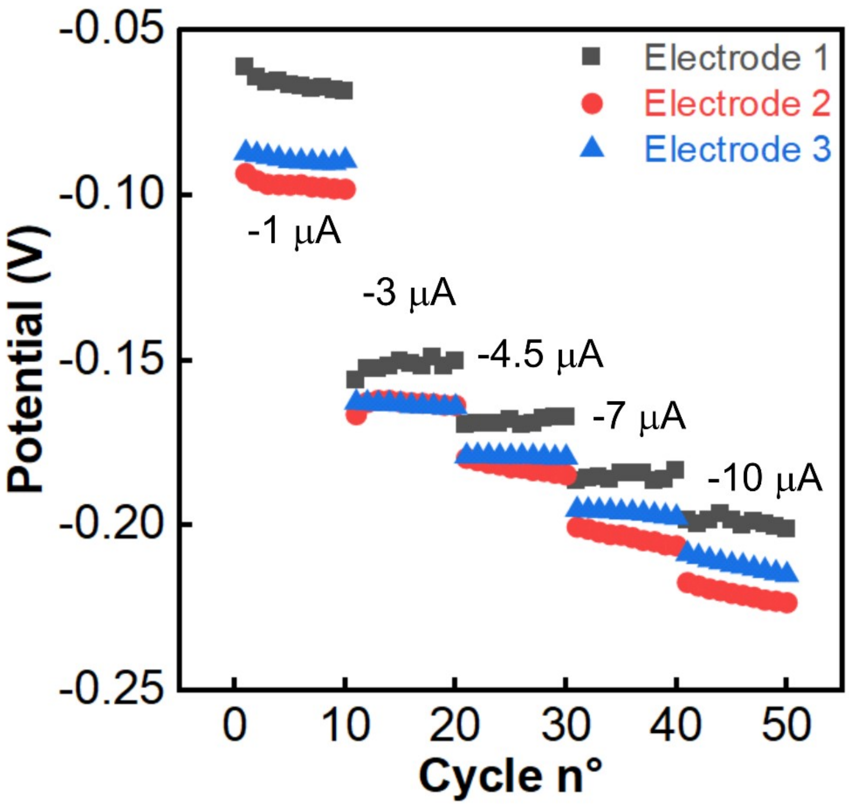

Figure S14. Study of the influence of variable current amplitude during step (I) on the stability of the reference pulses. Reference electrode: Silver/silver chloride double junction and counter electrode: Platinum. Background electrolyte: 0.15 M KNO_3.

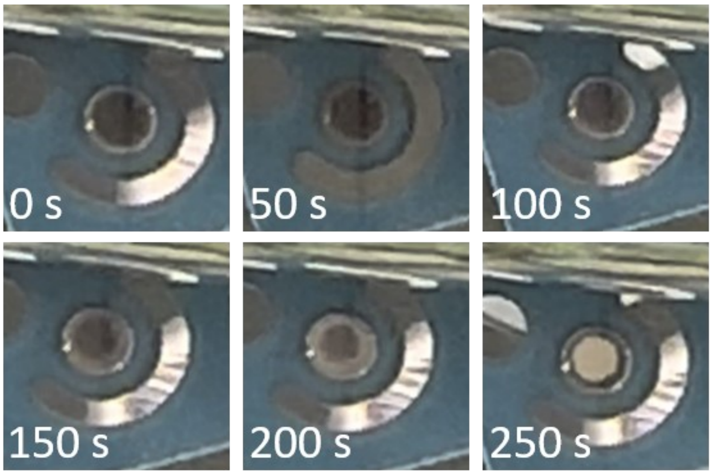

Figure S15. Images at different times of one inkjet-printed electrode previously deposited with the silver iodide layer, undergoing a cathodic pulse of -15.7 μA in 0.15 M KNO3.

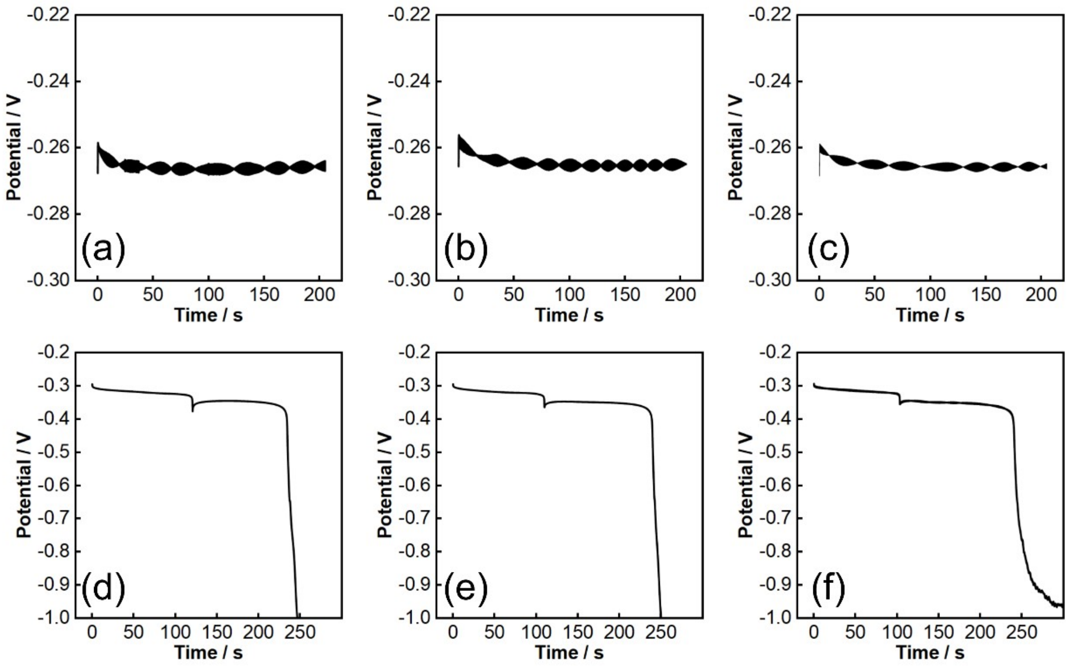

Figure S16. Potentiometric traces for three different electrodes under application of anodic [(a) to (c)] and cathodic [(d) to (f)] galvanostatic currents of respectively 15.7 and -15.7 μA in 0.1 M NaI solution. Reference electrode: Silver/silver chloride double junction and counter electrode: Platinum.

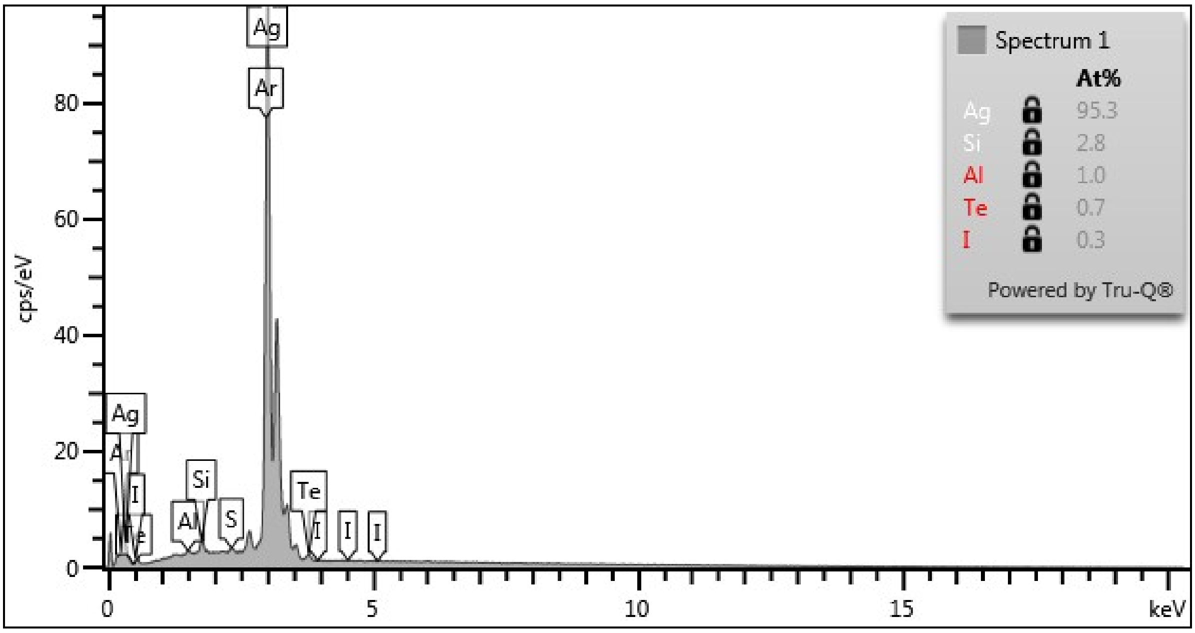

Figure S17. Energy Dispersive X-ray spectrum (voltage: 20 keV and current intensity: 8 A) of undeposited inkjet-printed electrode (bare silver).

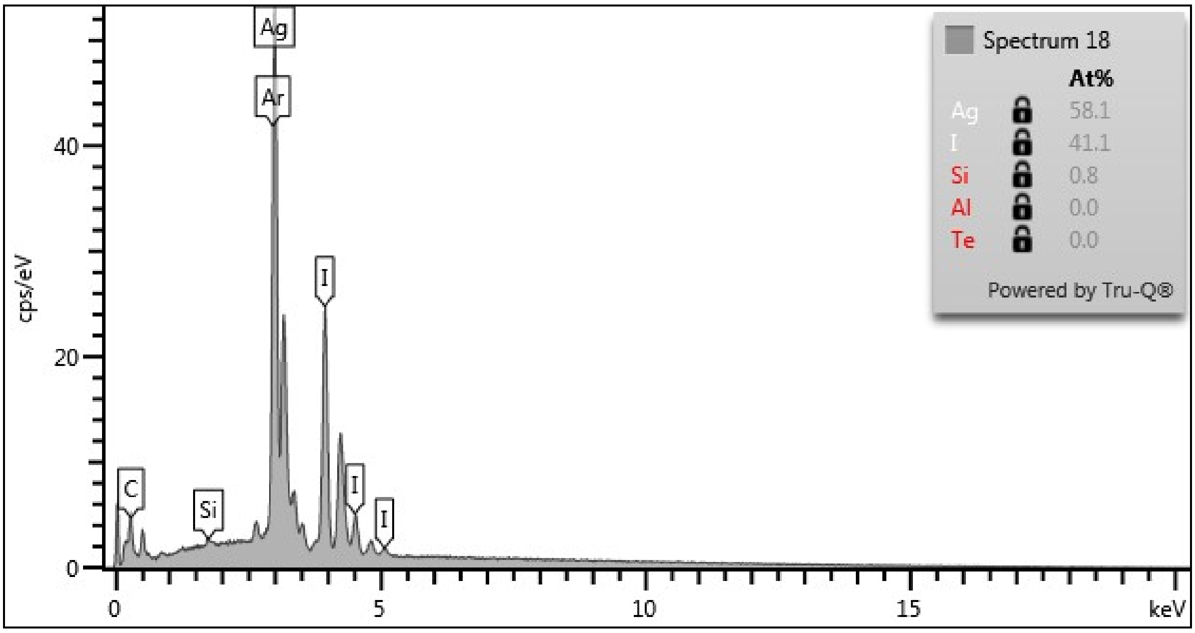

Figure S18. Energy Dispersive X-ray spectrum (voltage: 20 keV and current intensity: 8 A) of a freshly deposited inkjet-printed electrode.

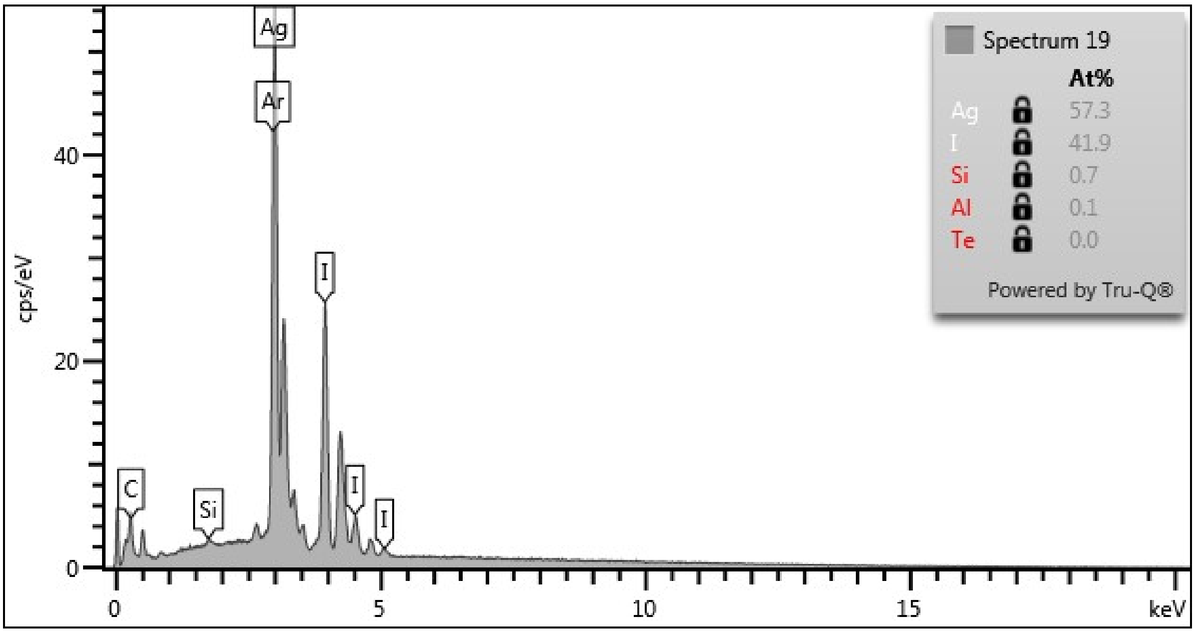

Figure S19. Energy Dispersive X-ray spectrum (voltage: 20 keV and current intensity: 8 A) of inkjet-printed electrode deposited 24 hours before analysis.

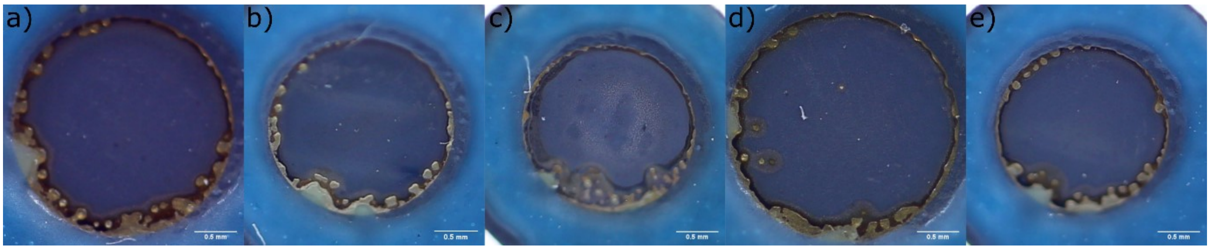

Figure S20. Images of five different inkjet-printed electrodes after deposition for 205s (current applied: 15.7 μA) in 0.1 M NaI solution. The images demonstrate the lack of reproducibility of the electro-active areas.

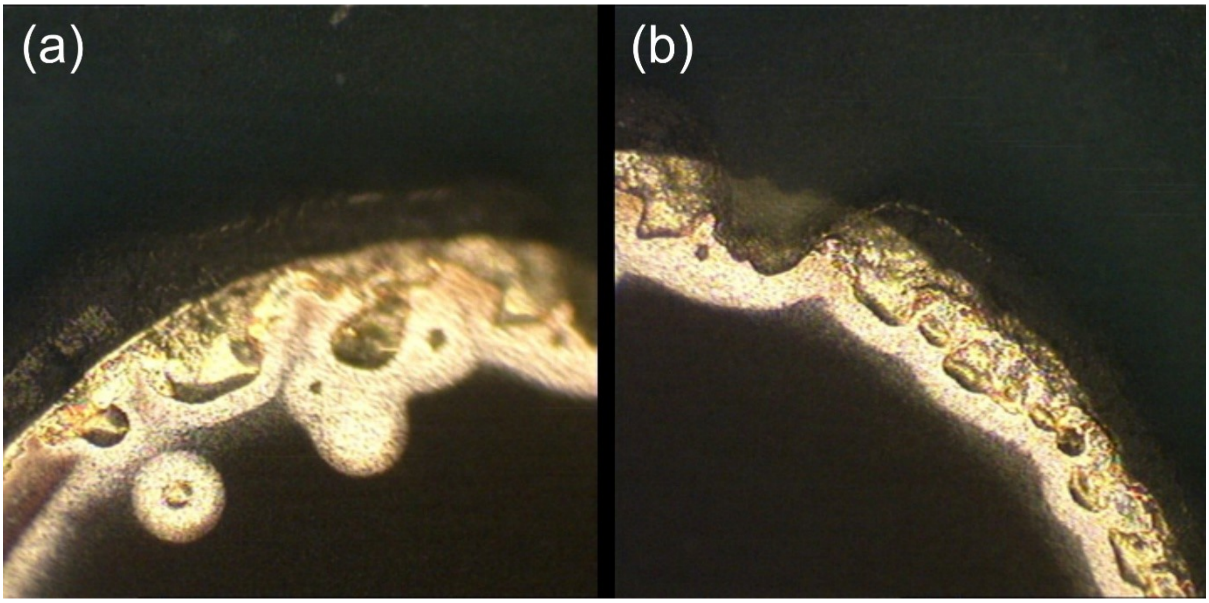

Figure S21. Micrographs (magnification: 10x) of two inkjet-printed electrodes (a) and (b) after deposition for 205 s (current applied: 15.7 μA) in 0.1 M NaI solution. The imaging clearly demonstrates the problematic leakage of dielectric onto the silver layer.

5

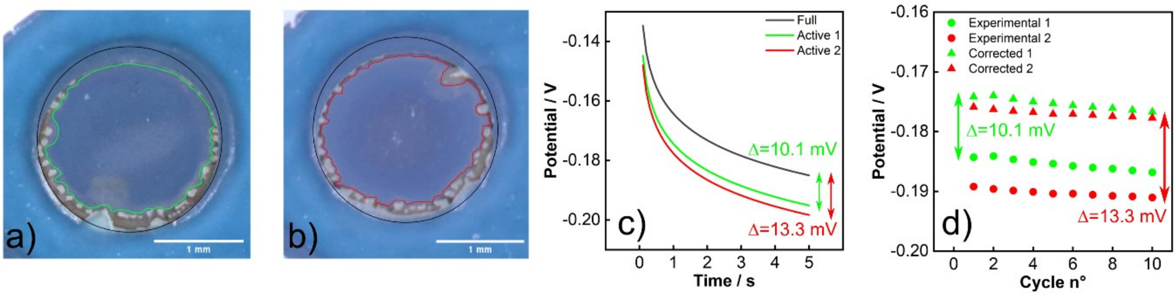

Figure S22. (a) and (b) quantification of the electro-active area of inkjet-printed electrodes. (c) expected potential during the release pulse based on different electro-active areas and (d) experimental and corrected reference potentials based on electro-active area quantification.

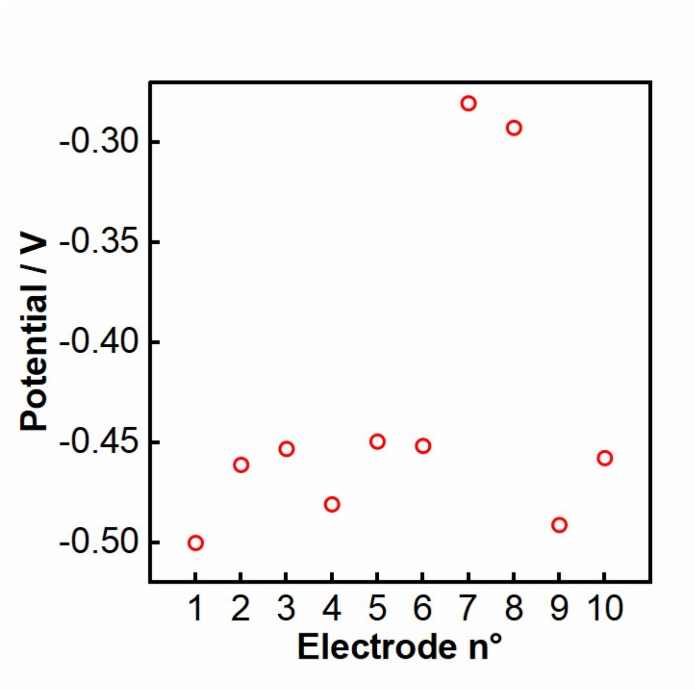

Figure S23. Reference pulses obtained after one cycle of pulstrode protocol in artificial urine for 10 different inkjet-printed electrodes, stored in the dark for 24 hours. Reference electrode: Silver/silver chloride double junction and counter electrode: Platinum. Parameters of the three steps (I) application of a current amplitude of -4.5 µA for 5 s, (II) detection for 0.25 s, (III) application of a regeneration potential equal the open circuit potential (OCP for 30 s.

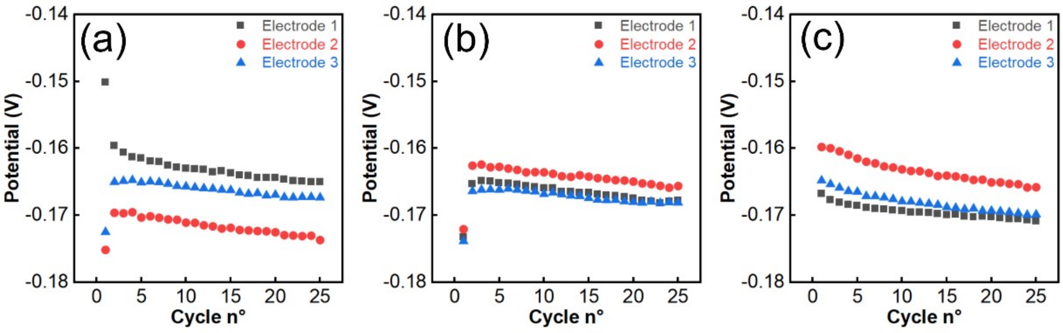

Figure S24. Study of the influence of storage conditions of the stability of the reference pulses for 25 consecutive cycles of pulstrode protocol. (a) Stored in the dark for 24 hours after deposition, (b) stored in light for 24 hours after deposition and (c) freshly deposited. Background electrolyte: KNO_3 0.15 M. Reference electrode: Silver/silver chloride double junction and counter electrode: Platinum. Parameters of the three steps (I) application of a current amplitude of -4.5 µA for 5 s, (II) detection for 0.25 s, (III) application of a regeneration potential equal the open circuit potential (OCP) for 30 s.

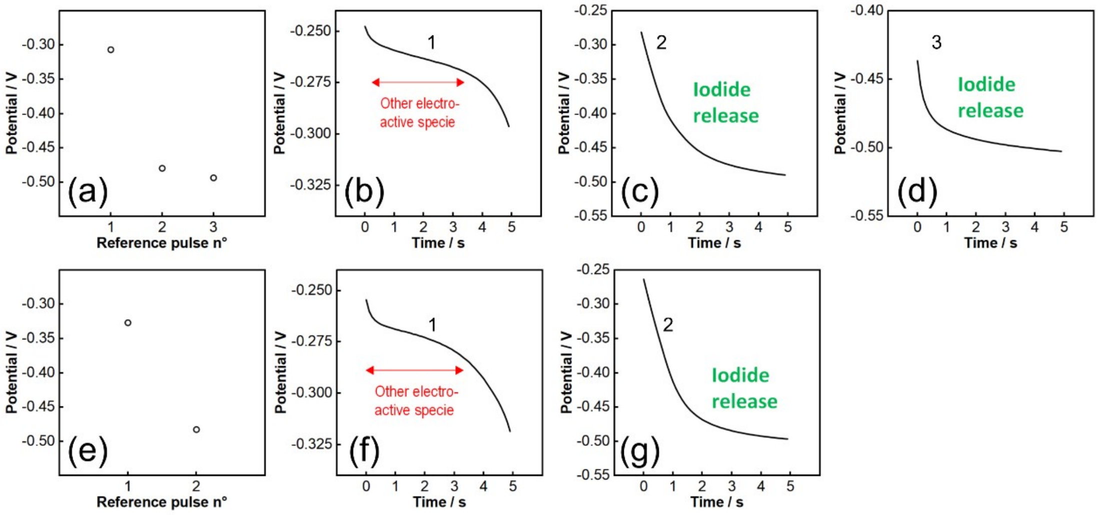

Figure S25. (a) and (e) references pulses in artificial urine for two different electrodes after storage for 24 hours in the dark. (b), (c), (d), (f) and (g): potentiometric traces of the corresponding release pulses at -4.5 µA. Reference electrode: Silver/silver chloride double junction and counter electrode: Platinum.

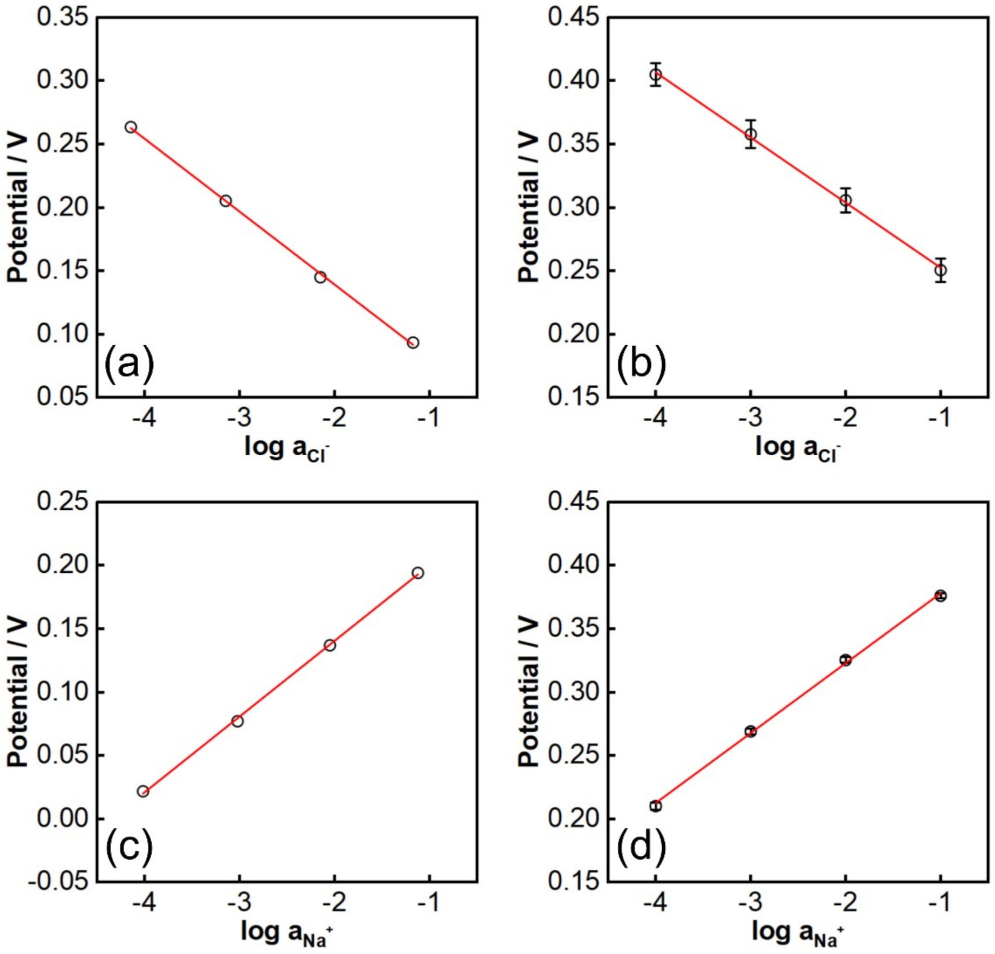

Figure S26. Observed potentiometric response to different activities of chloride [(a) and (b)] and sodium [(c) and (d)] respectively, using either the Ag/AgI based pulstrode protocol [(a) and (c)] and silver/silver chloride double-junction reference electrode [(b) and (d)].

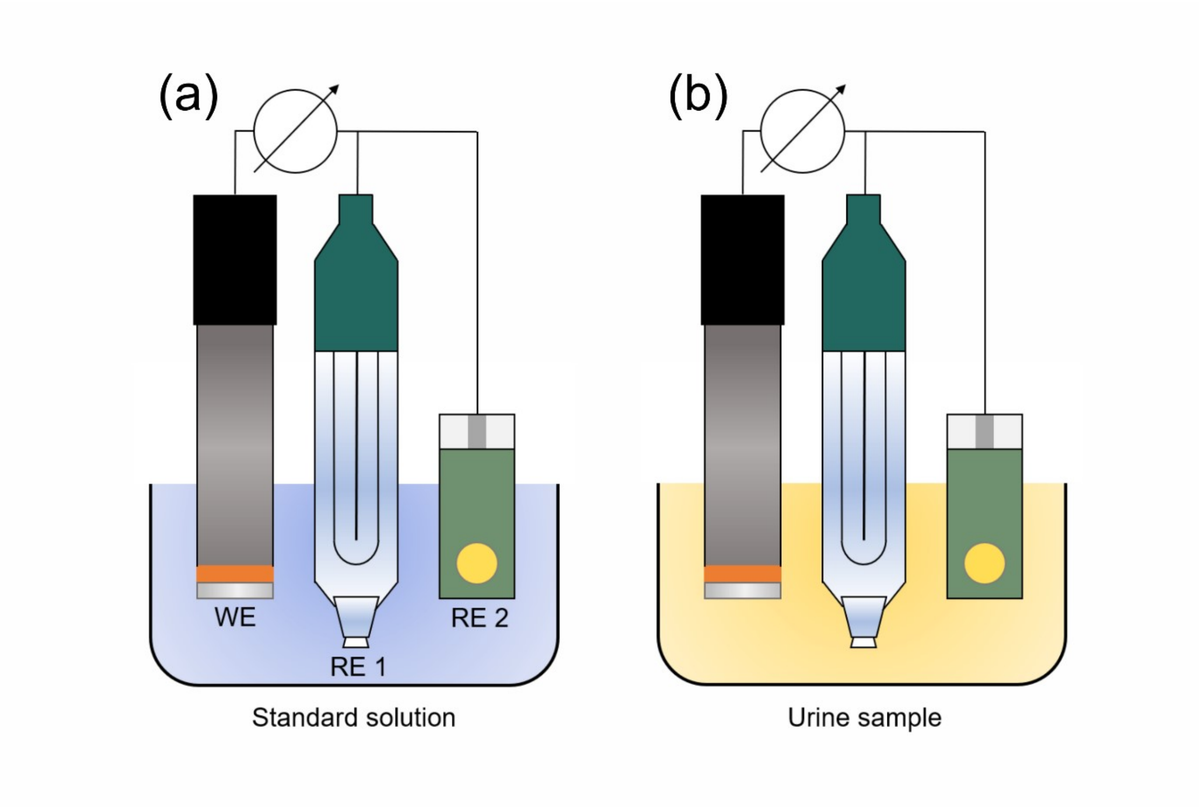

Figure S27. Schematic of the experimental design used for the quantification of ions in urine. The sensing component (WE) is either a plated Ag/AgCl, or a common polymeric membrane ion-selective electrode for sodium. The reference elements compared are the conventional double junction reference electrode (RE 1) and the inkjet-printed electrode provided by CSEM (RE 2). Counter electrode: platinum electrode. Using a first solution of synthetic urine which acts a standard solution of similar ionic strength, the system is calibrated. Assuming Nernstian behavior, the subsequent potential reading in real urine sample can be correlated to the concentration of the ions.

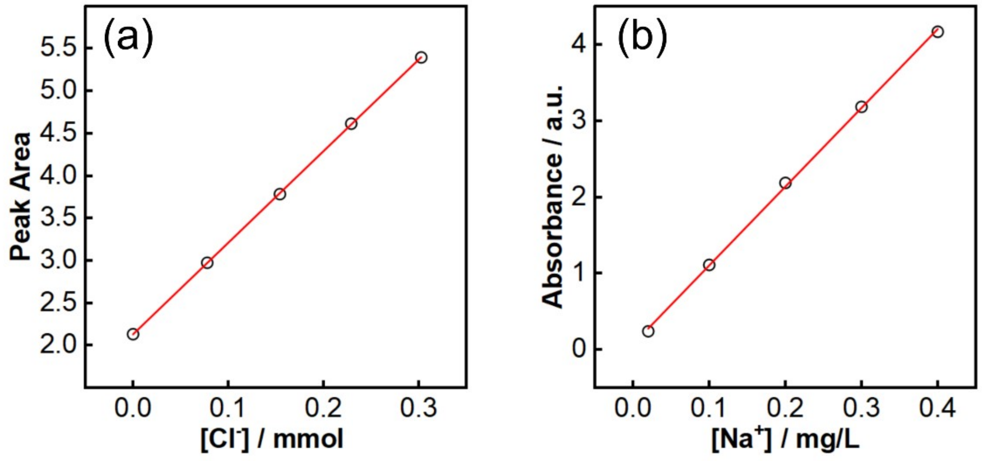

Figure S28. (a) Ion-Chromatography and (b) Atomic Emission Spectroscopy calibration curves used for the cross-correlation quantification of chloride and sodium in urine samples. Both techniques showed excellent correlation coefficient, with R^2 values of respectively 0.99996 (IC) and 0.99948 (AES).

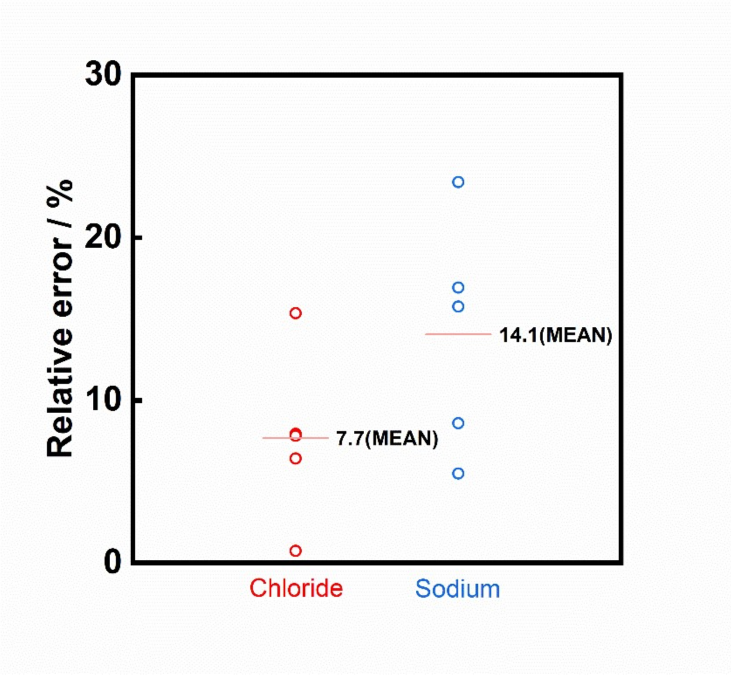

Figure S29. Errors of Inkjet-Printed electrodes (N=5 for each ion) for chloride and sodium urine measurements relative to the values obtained with a classical reference electrode.

Tables

Table 1. Energy Dispersive X-Ray (EDX) results for three different inkjet-printed electrodes. The atomic composition is expressed in percentage (%).

Element<br>Electrode type	**Al**	**Si**	**Ag**	**Te**	**I**
**Ag**	1	2.8	95.3	0.7	0.3
**AgI / Ag (fresh)**	0	0.8	58.1	0	41.1
**AgI / Ag (24 hours)**	0.1	0.7	57.3	0	41.9

Table 2. Experimental data, real current density and corrected potentials using a Mathematica model.

Electrode n°			
**1**		**2**	
**Full (dm^2)**	**Active(dm^2)**	**Full (dm^2)**	**Active(dm^2)**
3.141	2.120	3.141	1.872
**Calculated potential after release pulse for active area (V)**			
-0.1950		-0.1982	

Delta full-active (mV)	
10.1	13.3
